# Clinical validation of an AI-based blood testing device for diagnosis and prognosis of acute infection and sepsis

**DOI:** 10.1038/s41591-025-03933-y

**Published:** 2025-09-30

**Authors:** Oliver Liesenfeld, Sanjay Arora, Tom P. Aufderheide, Casey M. Clements, Elizabeth DeVos, Miriam Fischer, Evangelos J. Giamarellos-Bourboulis, Stacey House, Roger L. Humphries, Jasreen Kaur Gill, Edward Liu, Sharon E. Mace, Larissa May, Edward Michelson, Tiffany M. Osborn, Edward Panacek, Richard E. Rothman, Wesley H. Self, Howard A. Smithline, Jay Steingrub, Paul Van Heukelom, Alexandra Weissman, Matthew Wilson, Donna M. Wolk, David W. Wright, Ljubomir Buturovic, Yehudit Hasin-Brumshtein, Nandita Damaraju, Cici Lu, Joshua R. Shak, Natalie N. Whitfield, Purvesh Khatri, Timothy E. Sweeney, Nathan I. Shapiro

**Affiliations:** 1 Clinical Affairs, Inflammatix, Inc., Sunnyvale, CA USA; 2 Department of Emergency Medicine, University of Southern California, Los Angeles, CA USA; 3 Department of Emergency Medicine, Medical College of Wisconsin, Milwaukee, WI USA; 4 Department of Emergency Medicine, Mayo Clinic, Rochester, MN USA; 5 Department of Emergency Medicine, University of Florida College of Medicine-Jacksonville, Jacksonville, FL USA; 6 Department of Emergency Medicine, Georgetown University School of Medicine, Medstar Washington Hospital Center, Washington, DC USA; 7 4th Department of Internal Medicine, National and Kapodistrian University of Athens Medical School, Athens, Greece; 8 Department of Emergency Medicine, Washington University, St. Louis, MO USA; 9 Department of Emergency Medicine, University of Kentucky, Lexington, KY USA; 10 Department of Emergency Medicine, Henry Ford Hospital, Detroit, MI USA; 11 Department of Emergency Medicine, Hackensack Meridian Health, Jersey Shore University Medical Center HOPE Tower, Neptune, NJ USA; 12 Department of Emergency Medicine, Cleveland Clinic, Cleveland, OH USA; 13 Department of Emergency Medicine, University of California, Davis, Davis, CA USA; 14 Department of Emergency Medicine, Texas Tech University Health Sciences Center El Paso, El Paso, TX USA; 15 University of South Alabama, Mobile, AL USA; 16 Department of Emergency Medicine, Johns Hopkins University, Baltimore, MD USA; 17 Department of Emergency Medicine, Vanderbilt University Medical Center, Nashville, TN USA; 18 University of Massachusetts Chan Medical School-Baystate, Springfield, MA USA; 19 Department of Emergency Medicine, University of Iowa, Iowa City, IA USA; 20 Department of Emergency Medicine, University of Pittsburgh School of Medicine, Pittsburgh, PA USA; 21 Geisinger Diagnostic Medicine Institute, Geisinger Commonwealth School of Medicine, Danville, PA USA; 22 Department of Emergency Medicine, Emory University School of Medicine, Grady Memorial Hospital, Atlanta, GA USA; 23 Institute for Immunity, Transplantation and Infection, School of Medicine, Stanford University, Stanford, CA USA; 24 Center for Biomedical Informatics Research, Department of Medicine, Stanford University, Stanford, CA USA; 25 Department of Emergency Medicine, Beth Israel Deaconess Medical Center, Boston, MA USA; 26 Present Address: Institute of Microbiology, Infectious Diseases and Immunology, Charité – Universitätsmedizin Berlin, Berlin, Germany

**Keywords:** Bacterial infection, Viral infection

## Abstract

Lack of reliable diagnostics for the presence, type and severity of infection in patients presenting to emergency departments with non-specific symptoms poses considerable challenges. We developed TriVerity, which uses isothermal amplification of 29 mRNAs and machine learning algorithms on the Myrna instrument to determine likelihoods of bacterial infection, viral infection and need for critical care interventions within 7 days. To validate TriVerity, the SEPSIS-SHIELD study enrolled 1,222 patients with clinically adjudicated infection status and need for critical care intervention within 7 days as endpoints. The TriVerity Bacterial and Viral scores had higher accuracy than C-reactive protein, procalcitonin or white blood cell count for the diagnosis of bacterial infection with area under the receiver operating characteristic (AUROC) of 0.83, and viral infection (AUROC = 0.91). The TriVerity Severity score had an AUROC of 0.78 for predicting illness severity and allowed reclassification of risk for critical care interventions compared to clinical assessment (quick Sequential Organ Failure Assessment) alone. Each of the three scores had rule-in specificity >92% and rule-out sensitivity >95%. Comparison of antibiotics administration at presentation with post-follow-up adjudication found that TriVerity could potentially reduce false positives and false negatives for inappropriate antibiotics use by 60–70%. Further clinical testing in an interventional setting is needed to prove actionability and clinical benefit of TriVerity.

## Abstract (web summary)

In a prospective study enrolling 1,222 patients from 22 emergency departments, a device using a machine-learning-based signature of blood mRNAs demonstrated clinically acceptable performance to diagnose bacterial and viral infections and to predict the all-cause need for critical care interventions within 7 days, with benchmark to established biomarkers and risk scores.

## Main

Sepsis, an often fast-progressing and potentially fatal condition, is defined as a life-threatening acute organ dysfunction caused by a dysregulated host response to an infection^1^. Sepsis requires rapid administration of antimicrobials and fluids^2,3^ because the risk of death from sepsis continues to be high^4,5^. Sepsis is often subtle and difficult to detect, and the diagnosis is frequently missed, with potentially deadly consequences. As a result of difficult diagnosis and an imperative for early treatment, many patients with suspected sepsis who have alternative diagnoses are overtreated with antibiotics and hospitalization, which is costly, potentially harmful and contributes to antibiotic resistance. The need to determine whether a patient with suspected sepsis should be treated with anti-infective therapy (for example, antimicrobials and/or surgical source control) or not, and whether they should be hospitalized or not, underscore the need to assess a patient with suspected infection along two ‘axes’: infection presence and severity^6,7^.

There are currently no tests used in clinical practice that rapidly and reliably determine the presence and severity of infection^6,8^. Protein-based biomarkers, such as C-reactive protein (CRP) and procalcitonin, or direct pathogen detection tests can assist in the diagnosis of infection but often lack the accuracy and/or speed to allow identification of the presence of infection or to distinguish bacterial from viral etiology that is important for antibiotic decision-making at the time of presentation^9,10^. Patients with suspected infection (for example, belly pain) may in fact have a non-infectious cause of illness (pancreatitis); hence, a ‘sepsis’ test should also be able to diagnose whether a patient is infected or not. Although early admission to the intensive care unit (ICU) is imperative for patient survival advantages^11^, biomarkers (for example, lactate and cellular morphology tests) and clinical scores (for example, Sequential Organ Failure Assessment (SOFA) and quick SOFA (qSOFA)) for estimating risk can be time-consuming to collect and have limited accuracy for predicting the likelihood of decompensation in broader populations^12,13^.

We developed the TriVerity test to address this unmet need by determining (1) the likelihood of a bacterial infection, (2) the likelihood of a viral infection and (3) all-cause risk of mechanical ventilation, vasopressor use and/or new renal replacement therapy (RRT) within 7 days (‘ICU-level care’). TriVerity assigns a sample into one of five interpretation bands ranging from Very Low to Very High for each of the three likelihood scores using machine learning algorithms applied to semiquantitative measurements of 29 host immune mRNAs, which were previously reported as associated with infection status, type and severity^14–17^. Testing takes approximately 30 minutes using the cartridge-based Myrna instrument, with an operator hands-on-time of under 1 minute. The scores each fall into one of five interpretation bands ranging from Very Low to Very High.

Here we report the results of the SEPSIS-SHIELD study conducted for US Food and Drug Administration (FDA) clearance (obtained 10 January 2025; K241676) across 22 emergency departments in the United States and Europe. Building on previous observational studies^18–21^, we prospectively recruited 1,441 patients with suspected acute infection or suspected sepsis and evaluated whether TriVerity can (1) diagnose bacterial infections versus viral infections versus non-infectious mimics and (2) predict need for ‘ICU-level care’. We compared the accuracy of TriVerity to established diagnostic and prognostic biomarkers and performed a preliminary investigation of potential clinical utility for improving appropriate administration of antibiotics and prediction of sepsis.

## Results

**Fig. 1:**
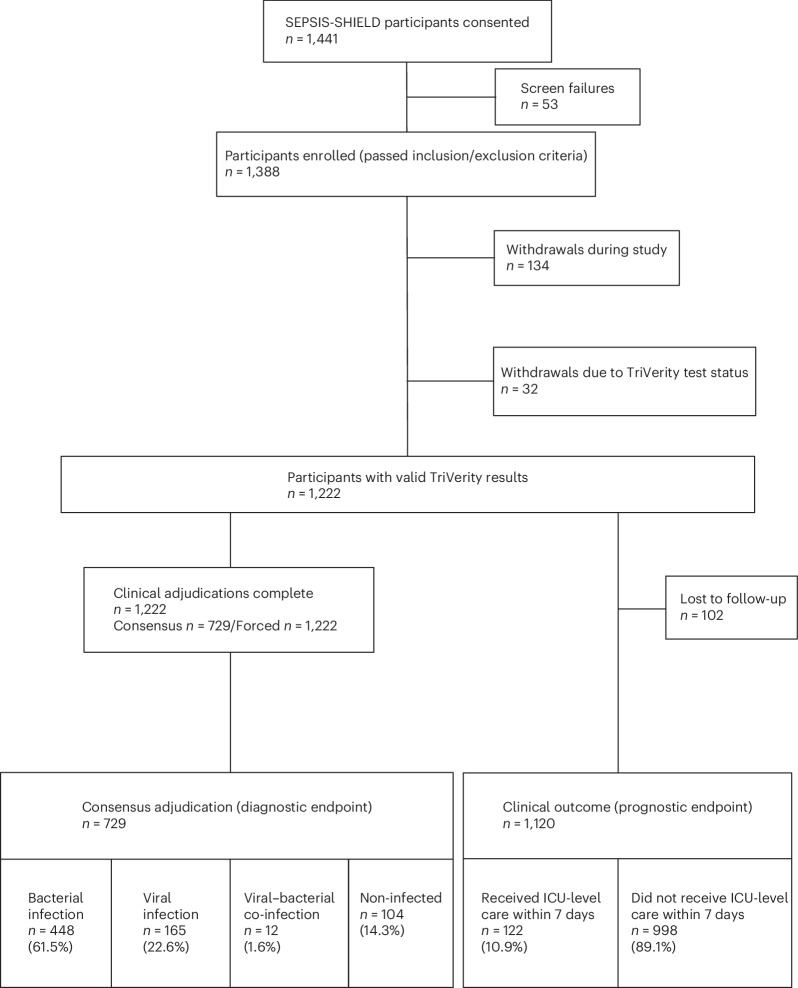
Overview of the study design and results for consensus clinical adjudication and clinical outcome.

Between March 2020 and May 2024, 1,441 adult patients with suspected acute infection or suspected sepsis and (1) at least one abnormal vital sign or (2) at least two vital sign changes with a blood culture order were enrolled from 22 emergency departments (Fig. 1 and Supplementary Table 1). After excluding screen failures and withdrawals, 1,222 patients had valid TriVerity results, and 729 of these were clinically adjudicated as consensus for the presence of a bacterial and/or viral infection (Yes or No adjudication status; Methods). We present these consensus-adjudicated patients as the main population (primary diagnostic endpoint); the secondary outcome of forced adjudication includes all 1,222 patients but with less certain adjudication status (Yes plus Probable and Unlikely plus No adjudications are grouped together; Methods). For the prognostic endpoint, 1,120 patients were evaluable (Fig. 1).

### Characteristics of study participants at emergency department presentations

Mean age, sex, race and ethnicity were representative of the US emergency department population (Extended Data Table 1). Among patients evaluable for the diagnostic endpoint, the mean age was 50.6 years, and 47.3% of the patients were female. Most patients were White (63.2%), followed by Black (30.6%) and Hispanic/Latino (13.3%). Similar percentages were observed among those evaluated for the prognostic endpoint (Extended Data Table 1). Metabolic/endocrinological, respiratory and cardiovascular diseases were the most prevalent medical conditions. There were no marked differences between patients evaluable for the diagnostic endpoint versus those evaluable for the prognostic endpoint. Overall, 132 patients (18.1%) evaluable for the diagnostic endpoint were immunosuppressed compared to 206 patients (18.4%) evaluable for the prognostic endpoint. Malignancies were the most frequently found type of immunosuppression (approximately 10% of patients), followed by solid organ transplantation, steroid treatment and HIV/AIDS (Extended Data Table 1).

The mean leukocyte counts in patients eligible for the diagnostic endpoint was 11.8 × 10^9^ per liter, the mean neutrophil percentage was 76.9 and the mean lymphocyte percentage was 12.5; similar numbers were observed in patients eligible for the prognostic endpoint (Supplementary Table 2a). Mean concentrations of biomarkers (CRP, procalcitonin and lactate) are shown in Supplementary Table 2b.

Among 1,120 patients eligible for the prognostic endpoint, 24.3% were discharged to home, 55.8% were admitted to a regular ward and 13.8% were admitted to an ICU (Extended Data Table 2a). The mean length of hospital stay was 5 days and of ICU stay was 6.1 days; there was no significant difference in consensus-adjudicated patients. Out of the 1,120 patients, 122 (10.9%) met the primary severity endpoint, which included mechanical ventilation (*n* = 63, 51.6%), vasopressor use (*n* = 99, 81.2%) and/or RRT (*n* = 23, 18.9%) (Extended Data Table 2b). Among 147 patients who were transferred to the ICU, 98 (66.7%) received the ‘ICU-level care’ interventions (composite primary severity endpoint). Extended Data Table 2b also shows differences in clinical outcomes stratified by clinically adjudicated infection status.

### Infection status and anatomical location of infection

Out of 729 consensus-adjudicated patients for diagnostic endpoint, 448 (61.5%), 165 (22.6%) and 12 (1.6%) had bacterial, viral and bacterial–viral co-infection, respectively, whereas 104 (14.3%) were adjudicated to not have an infection (Table 1). Among 460 patients adjudicated to have bacterial infections, urinary tract infections (*n* = 142, 30.9%) and skin or soft tissue infections (*n* = 133, 28.9%) were most frequent, followed by bloodstream (*n* = 92, 20%), gastrointestinal tract (*n* = 69, 15%) and respiratory tract (*n* = 58, 12.6%) infections. Among 177 patients adjudicated to have viral infections, 169 (95.5%) had respiratory tract infections, followed by gastrointestinal tract infections (*n* = 4, 2.3%). Three parasitic infections were diagnosed (*Giardia lamblia* enterocolitis, *n* = 1; *Trichomonas vaginalis* vulvovaginitis, *n* = 2). The percentage of bacterial infections was relatively high (63.1%), driven by seasonal epidemiology and the broad inclusion criteria allowing for enrollment of patients with all types of suspected infections (not only respiratory infections). Infection status for the forced adjudication cohort is shown in Supplementary Table 3a.

**Table 1:** Clinically adjudicated infection status and anatomical localization of infection

	Type of infection/anatomical location of infection^1,2^	% (*n*/*N*)
Type of infection	Bacterial infection	61.5% (448/729)
	Viral infection	22.6% (165/729)
	Viral–bacterial co-infection	1.6% (12/729)
	Non-infected	14.3% (104/729)
Bacterial adjudication	Bloodstream	20.0% (92/460)
	Central nervous system	0.4% (2/460)
	Gastrointestinal tract	15.0% (69/460)
	Joint	1.7% (8/460)
	Respiratory tract	12.6% (58/460)
	Skin or soft tissue	28.9% (133/460)
	Urinary tract	30.9% (142/460)
	Unknown/ Other	18.9% (87/460)
Viral adjudication	Bloodstream	0.6% (1/177)
	Central nervous system	1.1% (2/177)
	Gastrointestinal tract	2.3% (4/177)
	Respiratory tract	95.5% (169/177)
	Skin or soft tissue	1.1% (2/177)
	Unknown/ Other	3.4% (6/177)
Bacterial and/or viral adjudication	Bloodstream	12.8% (93/729)
	Central nervous system	0.5% (4/729)
	Gastrointestinal tract	10.0% (73/729)
	Joint	1.1% (8/729)
	Respiratory tract	31.1% (227/729)
	Skin or soft tissue	18.5% (135/729)
	Urinary tract	19.5% (142/729)
	Unknown/ Other	12.8% (93/729)

^1^Patients may have multiple sources.

^2^Anatomical localization of infection was determined by consensus clinical adjudicators after review of the clinical adjudication report.

### TriVerity result output

TriVerity provides three scores: Bacterial, Viral and Severity. Each score ranges from 0 to 50 and is divided into five interpretation bands (Very Low (0–10), Low (11–20), Moderate (21–30), High (31–40) and Very High (41–50)) that reflect increasing likelihoods of the corresponding infection type or severity. The suggested clinical interpretation of the two highest bands (‘Very High’ and ‘High’) is ‘rule-in’, whereas, for the two lowest bands (‘Very Low’ and ‘Low’), it is ‘rule-out’. Accuracy of TriVerity scores was evaluated as compared to post hoc clinical adjudications (Methods and ref. ^22^). For each of the three scores, when considering them separately, 80–86% of the patients were assigned to one of the rule-in or rule-out bands. When considered together, almost all of TriVerity results (99.6% and 99.3% of patients in the consensus and forced adjudication cohort, respectively) fell into one of the four clinically actionable interpretation bands (that is, Very Low, Low, High and Very High) for at least one of the diagnostic and prognostic scores.

### Accuracy of TriVerity for diagnosis of bacterial infection

The Bacterial score had an AUROC of 0.83 (80% CI: 0.81–0.85) for detecting bacterial infections in the consensus population. Probability of bacterial infection, as measured by likelihood ratio, ranged over 100-fold and increased monotonically by interpretation band (likelihood ratios: Very Low, 0.08 (80% CI: 0.05–0.11); Low, 0.54 (0.45–0.63); Moderate, 1.14 (0.94–1.42); High, 2.50 (1.97–3.31); Very High, 8.04 (5.66–12.43) (Table 2). The Bacterial score had specificity of 95.5% and 90.7% for the Very High and High bands, respectively, and sensitivity of 97.2% and 81.5% for the Very Low and Low bands, respectively (Table 2). Notably, 81.3% of the patients with consensus adjudication (and 80.4% of those with forced adjudication) fell into one of the clinically actionable Very Low, Low, High and Very High interpretation bands for bacterial infection. At a prevalence of 63.1% for bacterial infections, the probability of having a bacterial infection for the Very High interpretation band was 93.2% and for Very Low was 12.1%. Using forced adjudication (entire population including ‘uncertain’ adjudication—that is, probable and unlikely cases), the area under the curve (AUC) for the detection of bacterial infections was 0.76 (80% CI: 0.75–0.78) (Supplementary Table 3b); sensitivity, specificity and likelihood ratio for the accuracy of TriVerity Bacterial scores are shown in Extended Data Table 3a.

**Table 2:** Accuracy of TriVerity Bacterial score for the diagnosis of bacterial and viral infections

(a)							
TriVerity Bacterial band (score)	Clinically adjudicated bacterial infection^1^		Sensitivity (%)	Specificity (%)	Likelihood ratio (80% CI)	Relative requency of result (% in band)	Probability of bacterial infection (%)
	Yes (*N*)	No (*N*)					
Very High (40–50)	165	12	35.9	95.5	8.04 (5.66–12.43)	24.3	93.2
High (30–39)	107	25	23.3	90.7	2.50 (1.97–3.31)	18.1	81.1
Moderate (21–29)	90	46	19.6	82.9	1.14 (0.94–1.42)	18.7	66.2
Low (11–20)	85	92	81.5	34.2	0.54 (0.45–0.63)	24.3	48
Very Low (0-10)	13	94	97.2	34.9	0.08 (0.05–0.11)	14.7	12.1

(b)							
TriVerity Viral band (score)	Clinically adjudicated viral infection^1^		Sensitivity (%)	Specificity (%)	Likelihood ratio (80% CI)	Relative frequency of result (% in band)	Probability of viral infection (%)
	Yes (*N*)	No (*N*)					
Very High (40–50)	105	8	59.3	98.6	40.93 (27.73–72.16)	15.5	92.9
High (30–39)	25	33	14.1	94.0	2.36 (1.68–3.25)	8	43.1
Moderate 21–29)	22	79	12.4	85.7	0.87 (0.64–1.13)	13.9	21.8
Low (11–20)	17	166	90.4	30.1	0.32 (0.22–0.41)	25.1	9.3
Very Low (0–10)	8	266	95.5	48.2	0.09 (0.05–0.14)	37.6	2.9

^1^using consensus adjudication as the reference standard.

### Accuracy of TriVerity for the diagnosis of viral infection

The Viral score had an AUROC of 0.91 (80% CI: 0.89–0.93) for the detection of viral infections. Likelihood of viral infection increased monotonically more than 400-fold by interpretation band (likelihood ratios: Very Low, 0.09 (80% CI: 0.05–0.14); Low, 0.32 (80% CI: 0.22–0.41); Moderate, 0.87 (80% CI: 0.64–1.13); High, 2.36 (80% CI: 1.68–3.25) and Very High, 40.93 (80% CI: 27.73–72.16)). The Viral score had specificity of 98.6% and 94.0% for the Very High and High bands, respectively, and sensitivity of 95.5% and 90% for the Very Low and Low bands, respectively (Table 2). Notably, 86.1% of patients with consensus adjudication (and 81.3% of those with forced adjudication) fell into the clinically actionable Very High, High, Low and Very Low bands. At a prevalence of 24.3% for viral infections, the probability of having a viral infection in the Very High band was 92.9% and for Very Low was 2.9%. In the forced adjudication, the Viral score had an AUROC of 0.83 (0.81–0.85) for the detection of viral infections; sensitivity, specificity and likelihood ratio for the accuracy of TriVerity Viral scores are shown in Extended Data Table 3b.

The accuracy of the Viral score was robust in patients diagnosed with SARS-CoV-2 (Extended Data Table 4a), demonstrating its applicability and generalizability to emerging pathogens. Median Viral scores were highest in patients with infection from influenza A/B and SARS-CoV-2; patients diagnosed with human metapneumovirus and respiratory syncytial virus had intermediate Viral scores, whereas those with adenovirus and rhinovirus/enterovirus had the lowest Viral scores (Extended Data Table 4b).

Cross-classifications of both Bacterial and Viral scores for patients clinically adjudicated as bacterial infection, viral infection, co-infection and non-infected are shown in Supplementary Table 4a–d.

Overall, the Bacterial and Viral scores were strongly associated with increasing likelihoods of bacterial and viral infections, respectively. Notably, the Bacterial and Viral scores increased monotonically, and the 80% CIs for adjacent bands did not overlap in any of the analyses (Supplementary Fig. 1a,b).

### Accuracy of TriVerity compared to commonly used biomarkers

The AUROC of the Bacterial score (0.83, 80% CI: 0.81–0.85) was significantly higher than those of commonly used biomarkers for the diagnosis of infections, including procalcitonin (AUROC = 0.71, 80% CI: 0.68–0.73), CRP (AUROC = 0.74, 80% CI: 0.72–0.77) and white blood cell (WBC) counts (0.76, 80% CI: 0.73–0.78) (*P* < 0.0001 for all comparisons; Extended Data Table 5a). Supplementary Fig. 2 shows the correlation of WBC, CRP and procalcitonin concentrations with TriVerity Bacterial interpretation bands; Extended Data Table 2d shows the AUROCs for these biomarkers in the forced adjudication cohort.

In addition, the diagnostic accuracy of TriVerity generalized across races better than other biomarkers. Specifically, although the overall AUROC for procalcitonin was 0.71 for diagnosis of bacterial infection, it was substantially lower in Blacks (AUROC = 0.66) and other races (AUROC = 0.62) compared to Whites (AUROC = 0.74), highlighting lower clinical utility of procalcitonin in non-White populations. By contrast, the Bacterial score’s overall AUROC of 0.83 remained virtually identical in Whites (0.82) and Blacks (0.83) and was higher in other races (0.91) (Extended Data Table 5b).

Because TriVerity measures the host immune response to infection, we investigated whether the Bacterial and Viral scores maintained their diagnostic accuracy in immunocompromised patients, who also have an increased risk of infection. The AUROCs for TriVerity Bacterial and Viral scores were not significantly different between immunocompromised and immunocompetent patients (0.80, 80% CI: 0.75–0.85 versus 0.83, 80% CI: 0.81–0.85 for Bacterial scores; 0.89, 80% CI: 0.86–0.94 versus 0.91, 80% CI: 0.89– 0.94 for Viral scores) (Extended Data Table 6a,b). Lastly, when considering median Bacterial and Viral scores for specific anatomical sites of infection, patients adjudicated as positive for bacterial infection in the bloodstream had the highest median Bacterial score, followed by patients with bacterial infections of the respiratory and urinary tracts (Extended Data Table 7a,b). Only AUCs for the Bacterial scores in patients with bloodstream infections were significantly (*P* < 0.05) higher than the overall bacterial AUCs.

### Prognostic accuracy of the TriVerity Severity score

The Severity score predicted the need for ‘ICU-level care’, defined as an acute need for mechanical ventilation, vasopressor use and/or RRT within 7 days, with an AUROC of 0.78 (80% CI: 0.75–0.81). Risk of requiring ICU-level care monotonically increased by interpretation band over 50-fold (likelihood ratios: Very Low, 0.22 (80% CI: 0.14–0.31); Low, 0.43 (80% CI: 0.30–0.57); Moderate, 1.63 (80% CI: 1.35–1.97; High, 2.41 (80% CI: 1.96–2.91); Very High, 11.33 (80% CI: 7.07–17.75)). The Severity score demonstrated specificity of 98.7% and 86.1% for the Very High and High bands, respectively, and sensitivity of 91.8% and 87.7% for the Very Low and Low bands, respectively (Table 3). Most patients (79.6%) were in the clinically actionable Very High, High, Low and Very Low interpretation bands. At a prevalence of 10.9%, the probability of requiring ICU-level care within 7 days was 58.1% for the Very High band and 2.7% for the Very Low band. Likelihood ratios of the Severity scores for the three different individual components of ‘ICU-level care’ were similar to the overall likelihood ratios presented above (Extended Data Table 8). Kaplan–Meier survival analysis also found significantly increasing hazard ratios for the need of ‘ICU-level care’ between days 0 and 7 from ‘Very Low’ to ‘Very High’ Severity bands (Supplementary Fig. 3).

**Table 3:** Accuracy of TriVerity Severity score for the prediction of the need of mechanical ventilation, vasopressor use and/or RRT within 7 days

TriVerity Severity band (score)	Need for ‘ICU-level care’ within 7 days^1^		Sensitivity (%)	Specificity (%)	Likelihood ratio (80% CI)	Relative frequency of result (% in band)	Probability of severe illness (%)
	Yes	No					
Very High (40–50)	18	13	14.8	98.7	11.33 (7.07–17.75)	2.8	58.1
High (30–39)	41	139	33.6	86.1	2.41 (1.96–2.91)	16.1	22.8
Moderate (21–29)	38	191	31.1	80.9	1.63 (1.35–1.97)	20.4	16.6
Low (11–20)	15	288	87.7	28.9	0.43 (0.30–0.57)	27.1	5
Very Low (0–10)	10	367	91.8	36.8	0.22 (0.14–0.31)	33.7	2.7

^1^‘ICU-level care’: need for mechanical ventilation, vasopressor use and/or RRT within 7 days.

Lactate, a mandated biomarker commonly used to estimated severity as part of the SEP-1 bundle, had an AUROC of 0.76 (80% CI: 0.73–0.80) for predicting the need for ICU-level care. In the same patients, the Severity score had an AUROC of 0.78 (80% CI: 0.75–0.80). Lactate higher than 4 mmol l^−1^ demonstrated specificity of 95.8%, similar to the Very High and High bands for TriVerity Severity score. By contrast, sensitivity in patients with lactate lower than 2 mmol l^−1^ was 66.7% (Extended Data Table 9a), which was substantially lower than the sensitivity for Very Low and Low bands for the Severity score (>87%). In total, 213 patients had indeterminate lactate concentrations (2–4 mmol l^−1^), of whom 58 needed ‘ICU-level care’ or died (Extended Data Table 9b). Of these 58, TriVerity identified 46 (79.3%) as Moderate to Very High risk of severe illness, substantially reducing the uncertainty in identifying patients at higher risk of severe illness compared to lactate (Extended Data Table 9b). Hence, despite similar AUROCs, TriVerity demonstrated significantly lower false-negative and higher true-positive rates in patients with indeterminate lactate concentrations.

**Fig. 2:**
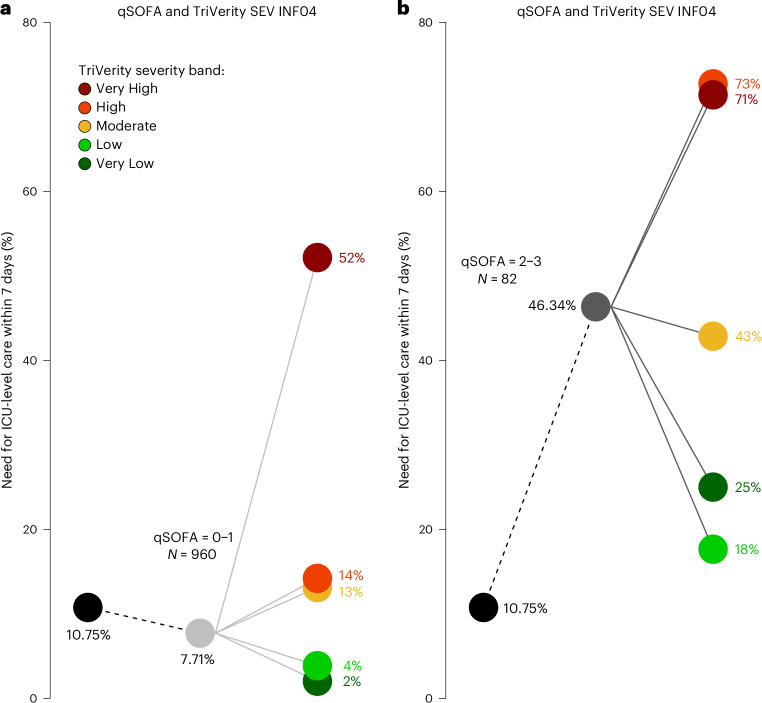
TriVerity Severity scores used in combination with qSOFA to predict the need for mechanical ventilation, vasopressor use and/or RRT (‘ICU-level care’) within 7 days. **a**, Patients with low qSOFA risk (scores 0–1). **b**, Patients with high qSOFA risk (scores 2–3). Overall percentages of patients with need for mechanical ventilation, vasopressor use and/or RRT (‘ICU-level care’) within 7 days independent of qSOFA and TriVerity results (= pre-test probabilities) are shown in black circles, whereas gray circles represent percentages of patients requiring ‘ICU-level care’ based on qSOFA scores alone. Colored circles represent the percent of patients requiring ‘ICU-level care’ based on combined qSOFA plus TriVerity Severity scores; colors represent TriVerity Severity score interpretation bands (Very Low, dark green; Low, bright green; Moderate, orange; High, bright red; Very High, dark red).

The severity of a patient’s clinical condition at the time of presentation in the emergency department can be assessed using clinical scores, such as the qSOFA score. We investigated whether integrating qSOFA with the TriVerity Severity score would further improve the accuracy of predicting ‘ICU-level care’. We determined pre-test and post-test probabilities of sequential qSOFA plus Severity scores (Fig. 2 and Supplementary Table 5). ICU-level care requirement among patients with low-risk qSOFA scores (0–1) was 7.7% and for those with high-risk qSOFA scores (2–3) was 46.3% (Fig. 2). However, stratifying patients by TriVerity Severity score interpretation bands markedly increased predictive accuracy in both the low and high clinical risk patients. For instance, a patient with qSOFA 0–1 (7.7% risk overall) would increase to a 52% risk with a ‘Very High’ TriVerity Severity score, whereas a patient with qSOFA 2–3 (46% risk overall) would decrease to a 18–25% risk with a ‘Low’ or ‘Very Low’ Severity score. The overall sensitivity of predicting ‘ICU-level care’ using qSOFA scores alone was 33.93%. When we combined qSOFA with the Severity score, the sensitivity increased significantly to 84.82% (*P* < 0.05).

Finally, the Severity score also predicted the composite need for ‘ICU-level care’ and/or 28-day mortality with a specificity of 99.0% for the rule-in Very High band and a sensitivity of 95.0% for the rule-out Very Low band, which further supports its predictive value of severe illness (Supplementary Table 6).

### Potential clinical utility

**Fig. 3:**
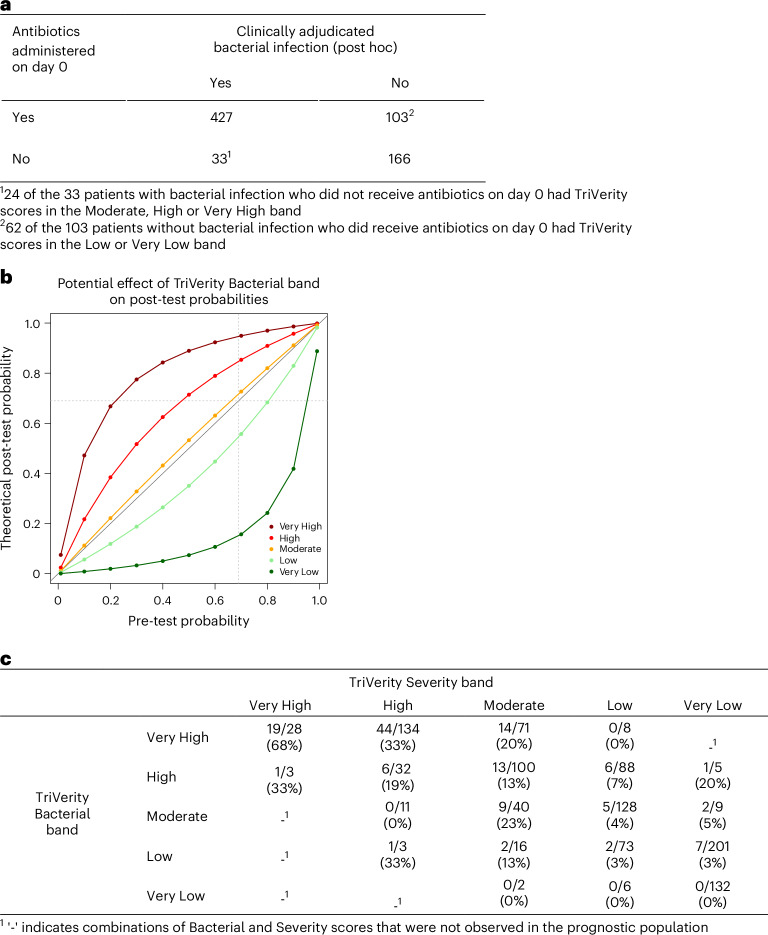
Potential diagnostic and prognostic clinical utility of TriVerity. **a**, Administration of antibiotics on day 0 compared to true infection status (post hoc clinically adjudicated bacterial infection). **b**, Post-test probabilities of bacterial infection as determined by pre-test probability and TriVerity likelihood ratios for the five Bacterial score bands (dark red, Very High; red, High; orange, Moderate; green, Low; dark green, Very Low). Pre-test and post-test probabilities for the five TriVerity Bacterial score bands are hypothetical but cover the entire range of possible probabilities. Dotted vertical and horizontal lines indicate physician decision thresholds (treat or not treat with antibiotics)^23^. **c**, Percent of patients in each combination of TriVerity Bacterial and Severity bands with bacterial sepsis, as by adjudicated bacterial infection and either delta in SOFA ≥ 2 or requirement of ICU-level care within 7 days.

To examine the potential clinical utility of TriVerity, we performed several preliminary analyses. We note that these analyses were not part of the preplanned analyses. First, we investigated whether the Bacterial scores could help reduce inappropriate antibiotic treatment using post hoc clinically adjudicated infection status as the gold standard (Fig. 3a). Thirty-three patients were adjudicated to have a bacterial infection but did not receive antibiotics on the day of presentation, of whom 10, 11 and 3 (overall 24 (72.7%)) had a Bacterial score of Moderate, High or Very High, respectively (Fig. 3a), suggesting that TriVerity Bacterial score could have helped emergency department providers avoid delays in antibiotic administration. On the other hand, 103 patients were adjudicated to not have a bacterial infection but received antibiotics on the day of presentation, of whom 62 (60.2%) had a Bacterial score of Low or Very Low (Fig. 3a), suggesting that TriVerity results could have helped emergency department providers avoid antibiotic overprescription.

Second, we applied likelihood ratios associated with TriVerity Bacterial bands to calculate theoretical post-test probability of bacterial infection for a range of hypothetical pre-test probabilities—ranging from 0 to 1 in 0.1-increment bins (Fig. 3b)—to reflect that physicians will use the Bacterial score in a variety of patients, including in those they judge infection is unlikely (for example, pre-test probability of 10%) or very likely (for example, pre-test probability of 90%). Using a threshold of 69% probability of bacterial infection for providers to prescribe antibiotics^23^, the post-test probability crosses the threshold in patients with ‘Very High’ Bacterial scores and pre-test probability as low as 0.3. Similarly, a ‘High’ Bacterial score results in a post-test probability of 0.69 in patients with a pre-test probability greater than 0.5. Conversely, Bacterial scores in the Very Low and Low bands reduce post-test probability below the 0.69 threshold for patients with a pre-test probability less than 90% and 80%, respectively (Fig. 3b). This analysis demonstrates that TriVerity Bacterial scores can assist emergency department providers in providing antibiotics to patients most likely to have bacterial infections and avoid antibiotics in patients less likely to have bacterial infections.

Finally, we explored whether the combination of high Severity and Bacterial scores would identify cases of bacterial sepsis. Because sepsis was not an adjudicated endpoint in the study, we used the definition of adjudicated bacterial infection and a change in SOFA score of ≥2 or requiring ICU-level care as a surrogate and examined the percent of patients with each combination of Bacterial and Severity score who met this definition for sepsis (Fig. 3c). We found that 68% of patients with a ‘Very High’ Bacterial score and a ‘Very High’ Severity score met this definition of sepsis. In addition, 33% of patients with a ‘High’ Bacterial score and a ‘Very High’ Severity score, or vice versa, met this definition of sepsis. In contrast, no patients with a ‘Very Low’ Bacterial score and a ‘Low’ or ‘Very Low’ Severity score had sepsis. Less than 3% of patients with a ‘Low’ Bacterial score and a ‘Low’ or ‘Very Low’ Severity score had sepsis.

Using a logistic regression model for infection status, including vital signs and laboratory values, and TriVerity (even though the adjudicators were blinded to all TriVerity results), the Bacterial score was the most significant variable associated with infection status, followed by WBC and procalcitonin (Supplementary Table 7).

## Discussion

Diagnosis and prognosis of patients presenting with symptoms of acute infections or suspected sepsis is an ongoing clinical challenge. Current diagnostic tools have limited sensitivity or require a long time to obtain results and do not assess prognosis or etiology^6,8,24^. Patients with suspected acute infection or suspected sepsis need to be evaluated along two clinical ‘axes’ (infection and severity) for appropriate treatment^6^. However, no single biomarker can adequately resolve a patient regarding presence (infected or not), type (bacterial or viral) and severity (risk of decompensation and organ dysfunction) of infection.

The SEPSIS-SHIELD study demonstrated high overall accuracy of the FDA-cleared TriVerity test, with sensitivities and specificities in the Very Low and Very High bands of 92–99% for establishing infection status and need for ‘ICU-level care’. This was true across multiple subpopulations and outperformed existing biomarker tests. Although a high proportion of patients were immunosuppressed, no significant differences were observed in the accuracy of TriVerity compared to immunocompetent patients. Notably, the performance in immunocompromised patients has not been determined in clinical studies for other host response tests, as these were validated in patient cohorts excluding immunosuppression^25^.

Our results suggest that TriVerity has potential to assist emergency department clinicians in several clinical decisions, including identifying patients who would benefit from anti-infective therapy, while mitigating antibiotic overuse, thereby assisting in reducing common side effects of antimicrobials and combating further emergence of antimicrobial resistance^26,27^. Notably, TriVerity met the expert consensus target product profile for diagnostic assays to differentiate between bacterial and non-bacterial infections for reducing antimicrobial overuse^28^. Recently introduced protein-based host response tests only distinguish bacterial versus non-bacterial etiologies but do not distinguish viral infections from non-infectious inflammation or co-infections^29,30^. By contrast, to our knowledge, TriVerity is the first direct ‘biomarker’ for the diagnosis of viral illness; specific and accurate identification of patients with viral infections will be adjunctive to other approaches for emergency department clinicians to decide on pathogen-specific viral diagnostics, antiviral treatment and isolation measures to prevent spread of infection. Furthermore, the simultaneous presentation of TriVerity Bacterial and Viral scores may reinforce the impact of a single bacterial result in support of antimicrobial stewardship. TriVerity Viral results were stable across different viruses, including influenza and SARS-CoV-2, although SEPSIS-SHIELD enrollment occurred before, during and after the COVID-19 pandemic. The virus-agnostic design and the high accuracy of TriVerity for the diagnosis of infection with SARS-CoV-2 also suggests its potential to assist in detection of emerging viral pathogens before pathogen-specific tests become available.

In addition, TriVerity also diagnosed bacterial–viral co-infections. Although the prevalence of co-infection is low in the emergency department, ruling out bacterial co-infections is important to allow withholding antibiotics, especially when radiographic or other clinical features introduce diagnostic uncertainty and during pandemic threats^31,32^. Finally, TriVerity results indicating low probabilities of bacterial and viral infection allow clinicians to focus their efforts on non-infectious diagnoses, thereby avoiding ordering of expensive pathogen identification tests.

The early identification and treatment of suspected sepsis is critical^33^, but there is disagreement among major societies regarding exact guidance and timing for optimizing clinical care. For example, the SEP-1 bundle from the Centers for Medicare & Medicaid Services and Surviving Sepsis Campaign guidelines strongly recommend the rapid administration of antibiotics to patients with suspected sepsis, but physician-based societies (for example, the Infectious Diseases Society of America) have argued that providers do not have adequate tools to judge who has sepsis, resulting in overtreatment and substantial costs associated with patients given unnecessary antibiotics^34,35^, including the rise of antimicrobial resistance. However, occult sepsis continues to be overlooked in a substantial proportion of emergency department patients. Although qSOFA ≥ 2 conveys a high mortality rate, further classification of patients with qSOFA ≤ 1 is needed because, despite low mortality rate, 30% of sepsis deaths occur in patients with low qSOFA^36^. In a post hoc analysis, after stratifying patients based on qSOFA, using TriVerity Severity score further improved the risk prediction dramatically by improving accuracy in those patients clinically at low risk but at substantially higher risk of severe illness. TriVerity also correctly identified those patients with substantially lower risk who had been clinically identified as high risk. The Severity score also provided additional information to aid in identification of patients with sepsis. An alternative test based on host mRNA expression was developed as an aid to differentiate infection-positive sepsis from infection-negative systemic inflammation but is cleared only for ICU use^37^. TriVerity also demonstrated high accuracy in ruling in and ruling out ICU transfer and predicted 28-day mortality with high accuracy. Taken together, these results suggest that TriVerity could improve patient management in the emergency department, with results offering potential for better informed clinical decision-making, including disposition decisions.

Taylor et al.^23^ recently surveyed 153 US clinicians responding to real-world derived vignettes of suspected sepsis to analyze decision thresholds to treat or not with antibiotics, using infection prediction models and varying case severities. Although the overall threshold to treat was 69% probability of infection, it varied significantly, such that it was 55% for high illness severity versus 84% for low illness severity. These findings strongly suggest the potential of the combined TriVerity diagnostic (Bacterial and Viral) and illness severity scores to further increase the threshold for antibiotic initiation. Incorporating context-dependent thresholds—potentially specific for TriVerity results in the outer Very High and Very Low bands versus the inner High and Low bands—into discriminating and well-calibrated models will inform the development of future clinical decision support systems.

Our preliminary results also demonstrate the potential clinical utility of the diagnostic and prognostic scores from TriVerity. In the SEPSIS-SHIELD study, TriVerity could have substantially reduced inappropriate antibiotic treatment by reducing both false positives (treated with antibiotics but no bacterial infection) and false negatives (bacterial infection not treated with antibiotics), which, in turn, could assist emergency department providers in acting as antimicrobial stewards. TriVerity also correctly identified patients who died without receiving the ICU-level care, demonstrating its potential to reduce the false negatives for prognosticating illness severity. The limitations of clinical estimation of infection and severity probabilities by emergency department providers reported here are in line with reports in the literature^24^. Further interventional studies are required to determine the clinical benefits of TriVerity.

Results of the present study, along with those of other studies^18–21^, support broad generalizability of our findings. Demographics of participants were representative of the emergency department patients with respect to age, sex, race and ethnicity, underlying conditions, immune status and clinical outcomes. Unlike procalcitonin, which has historically been less accurate in Black/African American and Hispanic subgroups^38^, TriVerity Bacterial scores maintained significantly higher accuracy than procalcitonin across White, Black and other races, suggesting its potential for improved diagnostic accuracy in minority populations. We also observed broad generalizability across patients with diverse suspected infection sites. Notably, we did not observe significant differences in the performance of TriVerity among patients with infections localized at different anatomical sites, except for bloodstream infections, which is expected because bloodstream infections would lead to stronger immune responses.

Notably, our statistical analysis approach ensured inclusion of all patients, as there was no equivocal adjudication (adjudication categories were Yes, Probable, Uncertain or No) and included all patients with results that fell into the ‘Moderate’ TriVerity bands. This approach is critical because several studies of other tests have been published in which statistical analysis excluded both patients with uncertain adjudication (a clinically critical ‘gray zone’ population) or uninfected and those in the middle (for example, equivocal) interpretation bands, thereby unfairly overstating those testsʼ overall accuracy^25,39–42^.

Our study has limitations. First, without a true gold standard, we used clinical adjudication as the reference standard to determine patient infection status^22^. As expected, the accuracy of the TriVerity test for diagnosing bacterial and viral infections was highest in the consensus population (certain infection status). Although consensus adjudication is the most accurate comparator to assess infection status, it excludes a fraction of patients with uncertain infection. As expected, including patients with uncertain infection status reduced the accuracy due to potential misclassifications in forced adjudication. Second, the prevalence of bacterial infections was higher than viral infections and non-infectious diseases, which was likely driven by (1) the broad inclusion criteria resulting in approximately 50% of all patients having abdominal, skin and soft tissue or urinary tract diseases (rarely caused by viral pathogens) and (2) the seasonality of bacterial versus viral infection. Depending on local epidemiology and setting, TriVerity may have varying positive and negative predictive values. However, we note that SEPSIS-SHIELD enrolled patients over cold and other seasons. Therefore, we think that the results reported here are unlikely to be affected by these factors. Furthermore, the development of the machine-learning-based TriVerity classifiers was based on a large number of datasets from highly diverse cohorts of patients with suspected infections and/or suspected sepsis^43^. Lastly, the percentage of patients who required ICU-level care was lower than expected; thus, although results of the TriVerity Severity score indicate high accuracy for the prediction of ‘ICU-level care’, future validation studies are warranted prior to use in substantially sicker populations. We previously showed consistently high accuracy of the TriVerity Severity score among patients admitted to the ICU^44–46^.

In conclusion, TriVerity rapidly detects bacterial infections, viral infections and bacterial–viral co-infections, can rule out bacterial and viral infections and has superior accuracy compared to routine biomarkers. TriVerity can also predict the severity of illness and, when combined with clinical scores, adds granularity and potential clinical utility for predicting severe infection, including sepsis. Interventional studies are needed, and are currently underway, to provide evidence of actionability and clinical benefit of TriVerity. These will determine whether TriVerity can enable personalized management of patients with suspected acute infections and suspected sepsis for improved overall healthcare outcomes.

## Methods

### Ethics statement

This study was approved by local institutional review boards (enrolling sites) or a central institutional review board (Advarra). Written consent was obtained from each patient. The study was conducted with the highest respect for the individual patients and in accordance with the protocol; the ethical principles originating from the Declaration of Helsinki; the informed consent regulations stated in Title 21 Code of Federal Regulations, Part 50; International Council for Harmonization of Technical Requirements for Pharmaceuticals for Human Use; Good Clinical Practice (E6) §4.8; and all applicable local and FDA regulations.

### Study design

Patients were enrolled in the ‘TriVerity in the Diagnosis and Prognosis of Emergency Department Patients with Suspected Infections and Suspected Sepsis’ (SEPSIS-SHIELD; NCT04094818) study, a prospective, non-interventional, minimal-risk study enrolling adult patients presenting to the emergency department with (1) suspected acute infection and at least one abnormal vital sign or (2) suspected sepsis with at least two vital sign changes and a blood culture order. Patients were enrolled from 22 emergency departments (Fig. 1 and Supplementary Table 1). Emergency departments of community and academic hospitals were located at 21 geographically diverse locations throughout the United States and at one site in Europe (Supplementary Table 1). During the ‘FROZEN’ phase of the study (2 March 2020 to 16 February 2023), blood samples were frozen and sent to one of two reference laboratories to perform TriVerity after thawing the frozen samples. During the ‘FRESH’ phase of the study (8 December 2023 to 28 May 2024), TriVerity was performed on freshly obtained blood samples (no freezing) at the enrolling site. Fresh–frozen equivalency was established by comparing the accuracy between results obtained in the FRESH phase versus those in the FROZEN phase of the study (Supplementary Fig. 4).

The study plan, conduct and statistical analysis were guided by detailed discussions with the FDA via the Q-Submission Program and in sprint discussions under the FDA’s Breakthrough Devices Program. Power analyses were based on performance characteristics of TriVerity (bacterial, viral and illness severity) and estimated prevalences of infections, non-infectious differential diagnoses and severe outcomes.

### Patients and sample and data collection

Inclusion criteria were as follows:

1. age ≥18 years
2. suspected acute infection (for example, respiratory, urinary, abdominal, skin and soft tissue infection, meningitis/encephalitis or any other infection)
3. at least one vital sign change (heart rate >90 beats per minute, temperature >38 °C or <36 °C, respiratory rate >20 breaths per minute or PaO_2_ <60 mmHg or SpO_2_ < 90%, systolic heart pressure <100 mmHg, altered mental status per clinical examination) or suspected sepsis of any cause (defined as blood culture order by the treating physician and at least two vital sign changes)
4. able to provide informed consent or consent by a legally authorized representative

Exclusion criteria were as follows:

1. Patient-reported treatment with systemic antibiotics, systemic antiviral agents or systemic antifungal agents within the past 7 days prior to the emergency department visit (use of antiviral treatment for HIV and hepatitis B and C, topical antibiotics, topical antivirals or topical antifungal agents, anti-herpes prophylaxis, perioperative (prophylactic) antibiotics and a single dose of antimicrobials during the present emergency department visit (<10 hours before blood draw) did not result in exclusion)
2. patients receiving palliative or hospice care or those receiving limited interventional care
3. prisoners, those mentally disabled or those unable to give consent
4. patients receiving experimental therapy or already enrolled in an interventional clinical trial in which a patient received some type of intervention, which could include, but was not limited to, investigational drugs, medical devices or vaccines
5. patients previously enrolled in this clinical trial

Of note, patients with any form of immunosuppression were eligible for SEPSIS-SHIELD.

All patients had routine samples collected as per the standard of care and were managed as per standard of care independent of the study conduct. The study intervention consisted of blood collection via venipuncture (2.5 ml of whole blood into PAXgene Blood RNA Tubes (PreAnalytix) for TriVerity testing and 5 ml of whole blood for central laboratory testing of serum CRP and procalcitonin) and a nasopharyngeal swab collection from patients with suspected upper respiratory tract infections. Patients were followed for up to 28 days; those discharged earlier were contacted via phone call to collect follow-up information (readmission, etc.).

Data collection using a case report form occurred from the day of presentation in the emergency department (covering 7 days before enrollment) to the follow-up phone call after day 28. Data collected included detailed information on demographics; medical history; clinical, laboratory and imaging findings; and information related to management, including treatment with antibiotics and ICU-level treatment. Furthermore, at the time of enrollment, a questionnaire was presented to the emergency department provider and was completed no later than 24 hours after the time of enrollment. The questionnaire included questions on the provider’s presumptive diagnosis, the provider’s assessment of probability of bacterial and viral infection as well as the need for ‘ICU-level care’. All data were stored pseudonymized in a secure database (Medrio).

### Endpoints

The primary endpoints for bacterial and viral etiology were determined by independent adjudicators following a transparent and standardized clinical adjudication process developed for the SEPSIS-SHIELD trial by a multidisciplinary team of physicians and laboratorians^22^. Adjudicators were blinded to the TriVerity results at all times and did not adjudicate cases enrolled at their own institution. In brief, two independent physicians (randomly chosen from a pool of 12 emergency department physicians) reviewed comprehensive clinical, laboratory and other patient information to adjudicate the presence or absence of bacterial and viral infections into Yes, Probable, Unlikely and No categories (no equivocal adjudication allowed). Discordant cases were resolved by one of two expert physicians (experienced and involved in generating the adjudication protocol) who were blinded to the initial reviewer results. Cases adjudicated as Yes and No (the subgroup with certain infection status) formed the consensus cohort presented in the main body of this report. Cases adjudicated as Probable or Unlikely were forced into categories of Yes/Probable and No/Unlikely to form the forced cohort (all patients including uncertain and certain adjudications). Results for the forced adjudication analysis are presented in Extended Data Table 2a–d. The primary severity outcome (patients requiring ‘ICU-level care’) was defined as receipt of acute mechanical ventilation, vasopressor and/or new RRT within 7 days. Admission to the ICU was captured independently.

### TriVerity test

**Extended Data Fig. 1:**
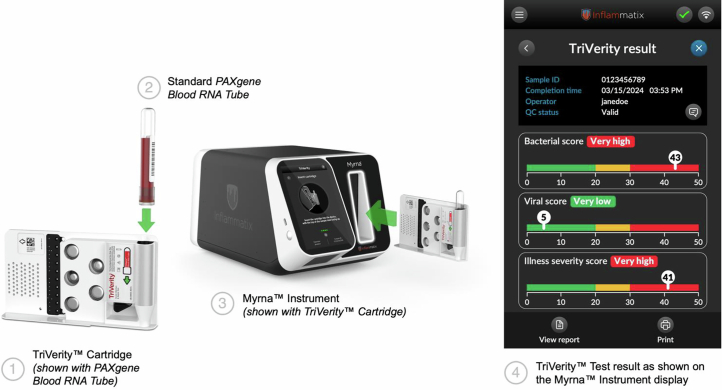
Components of TriVerity system. TriVerity System consists of a TriVerity cartridge, Myrna instrument and TriVerity test result readouts.

TriVerity (formerly HostDx or InSep) is a gene expression profiling assay that quantifies the relative expression of 29 host response genes from 2.5 ml of whole blood collected in PAXgene Blood RNA Tubes. The TriVerity system (Extended Data Fig. 1a and Supplementary Fig. 5a) comprises the TriVerity cartridge and the Myrna instrument with embedded software and proprietary preprocessing and machine learning classification algorithms that process expression data and deliver result readouts. In brief, the 29 mRNAs were selected from extensive multicohort analyses of thousands of blood transcriptome profiles across tens of independent cohorts to diagnose presence^16^, type^15^ and severity^17^ of infection and were further optimized^19^. On the Myrna device, the signal processing estimates 29 relative gene expression values based on the cartridge well camera images taken at regular intervals during loop-mediated isothermal amplification^47^. We apply two logistic regression classifiers to the mRNA expression data to estimate probabilities of bacterial, viral or no infection and the probability of severe illness. These classifiers (described in detail in ref. ^43^) were finetuned using a combination of hyperparameter optimization and grouped cross-validation approaches, which was found to reduce overfitting during a classifier development. The system is designed for single-sample testing. TriVerity converts probabilities to the three scores corresponding to (1) the likelihood of a bacterial infection, (2) the likelihood of a viral infection and (3) the severity of the patient’s illness. They fall into actionable interpretation bands ranging from Very High to High, Moderate, Low and Very Low (Extended Data Fig. 1b). TriVerity received FDA clearance on 10 January 2025. Results of analytical studies, including limit of detection, equivalency of fresh versus frozen samples, interfering substances, quality controls and other details of the TriVerity system, are described in the FDA Decision Summary (https://www.accessdata.fda.gov/scripts/cdrh/cfdocs/cfpmn/pmn.cfm?ID=K241676) and in a separate report^48^.

### Statistical methods

The analysis population consisted of all patients who provided consent, met all inclusion criteria, met no exclusion criteria and had PAXgene blood samples successfully processed (resulting in three TriVerity scores). The overall analysis population was divided into diagnostic and prognostic analysis populations. The diagnostic endpoint population included patients who had been adjudicated for bacterial and viral infection to define the ground truth, defined as either a certain (Yes or No) or an uncertain (Probable or Unlikely) infection status. The diagnostic population was further classified as Consensus (only contains patients with a certain (Yes or No) adjudication) and Forced (all patients adjudicated as (Yes or Probable) versus (No or Unlikely)). The primary prognostic endpoint population included patients with complete information regarding the presence of acute need for mechanical ventilation, vasopressor use and/or RRT within 7 days. The secondary prognostic endpoint included patients who met the primary endpoint and/or 28-day mortality.

TriVerity bands were assessed in terms of likelihood ratio, sensitivity, specificity and predictive values (post-test probabilities) dependent on the band (whether intended as a rule-in band with a case being a true positive or a rule-out band with a case being a false negative). For continuous variables (for example, age and WBCs), results were summarized with the numbers of observations, means, standard deviations, medians and ranges and 80% CIs. These calculations and endpoints were determined in discussions with the FDA and were used in prior FDA registrational clinical studies (https://www.accessdata.fda.gov/cdrh_docs/reviews/K210254.pdf). Fisher’s exact test determined significance for differences in need of ‘ICU-level care’ across patients as well as tested if there were significant differences in positive rate for each of the TriVerity Severity bands stratified by qSOFA scores. *P* values for differences between the AUROCs of TriVerity and biomarker values, clinical scores (qSOFA) and differences across races were calculated using DeLong’s test; *P* values comparing immunocompromised and immunocompetent patients as well as COVID-19 versus non-COVID-19 patients were calculated using bootstrapping. Statistical analyses were performed in SAS software version 9.4 or R software version 4.2.

### Reporting summary

Further information on research design is available in the Nature Portfolio Reporting Summary linked to this article.

## Online content

Any methods, additional references, Nature Portfolio reporting summaries, source data, extended data, supplementary information, acknowledgements, peer review information; details of author contributions and competing interests; and statements of data and code availability are available at 10.1038/s41591-025-03933-y.

## Supplementary information

Supplementary Information Supplementary Tables 1–7 and Supplementary Figs. 1–4.

Reporting Summary

## Figures and Tables

**Extended Data Table 1:**
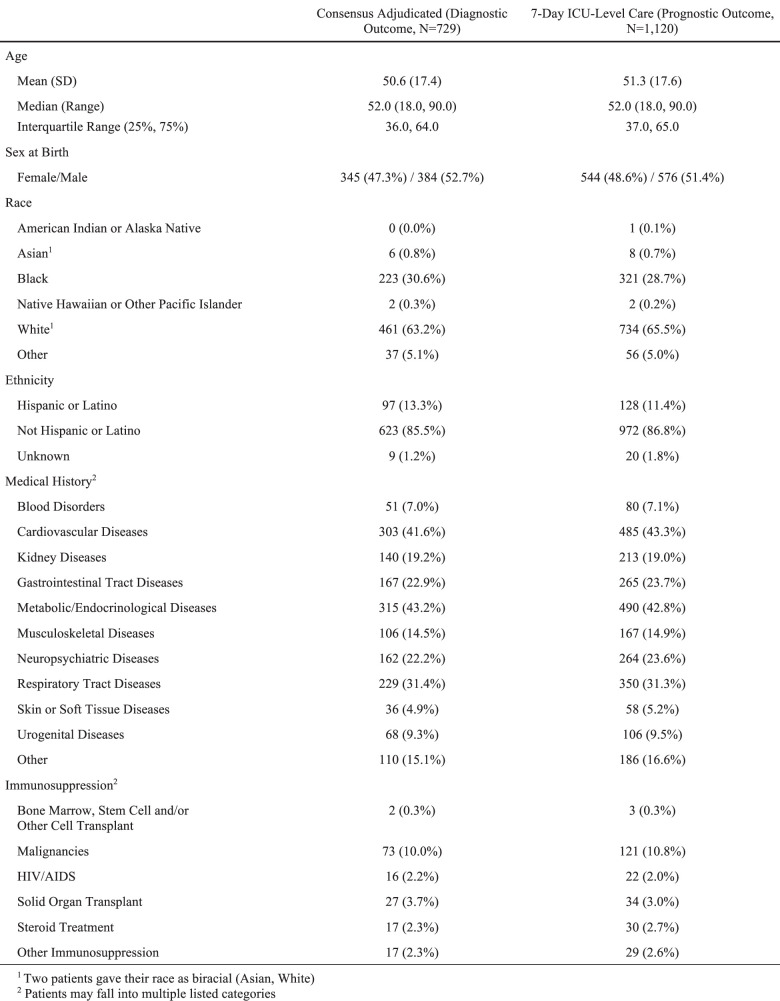
Overview of demographics and medical history of patients evaluable for the diagnostic and prognostic endpoint. ^1^ Two patients gave their race as biracial (Asian and White). ^2^ Patients may fall into multiple listed categories

**Extended Data Table 2:**
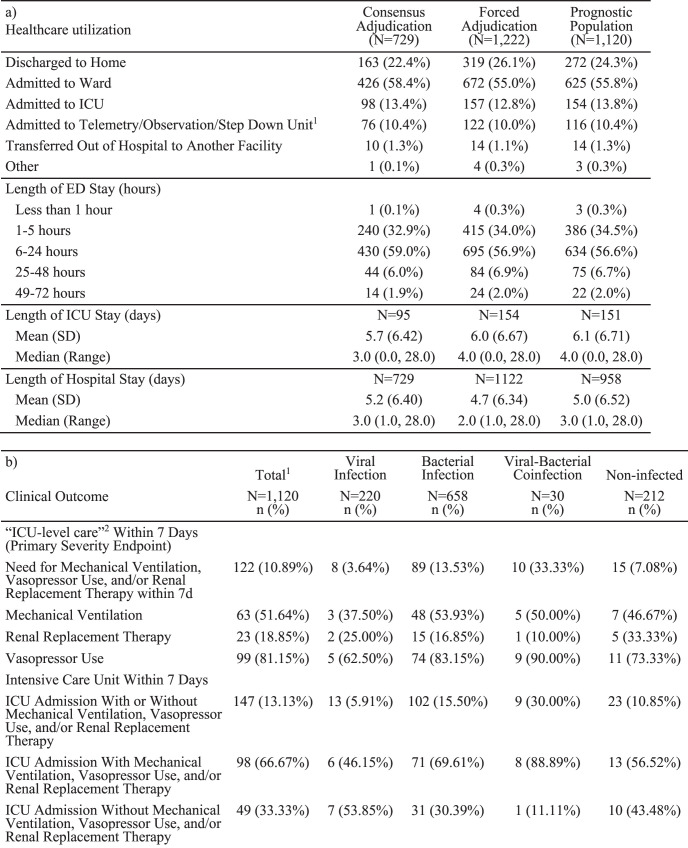
Healthcare use by patient population (**a**) and clinical outcomes (**b**). **a**, ^1^ Telemetry: patients requiring continuous cardiac monitoring. Observation unit: patients observed for up to 48 hours requiring additional hospital care but not meeting the criteria for admission to the hospital, often adjacent to and managed by the emergency department. Step-down unit: patients no longer needing ICU-level care but still more attention than those on a general ward. General ward: typical medical or surgical units. **b**, ^1^ Results shown are for the prognostic population (*n* = 1,120). ^2^ Defned as the need for mechanical ventilation, vasopressor use and/or RRT

**Extended Data Table 3:**
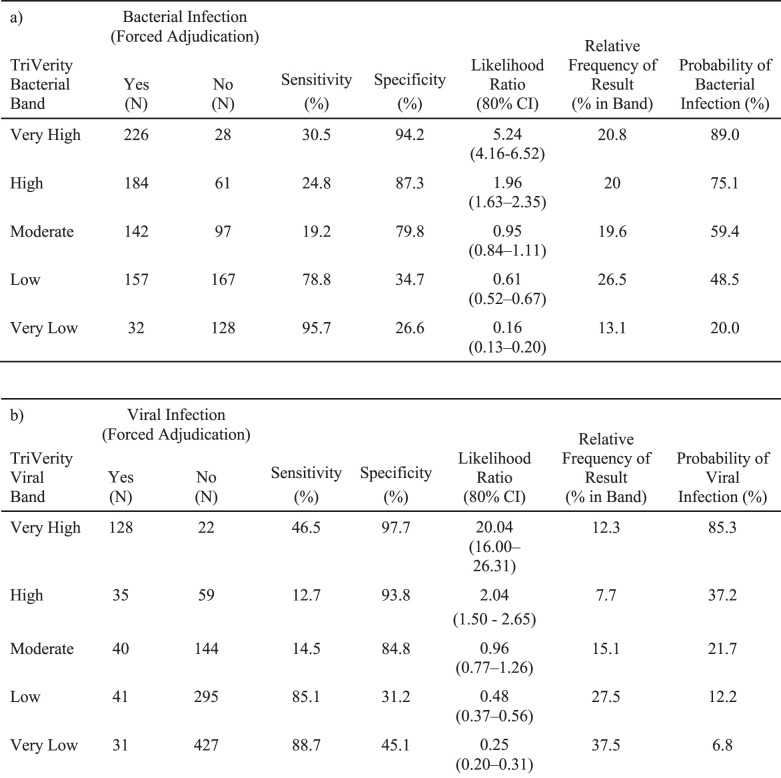
Accuracy of the TriVerity test in patients with forced adjudication (entire population including ‘uncertain’ cases adjudicated as probable and unlikely infected)

**Extended Data Table 4:**
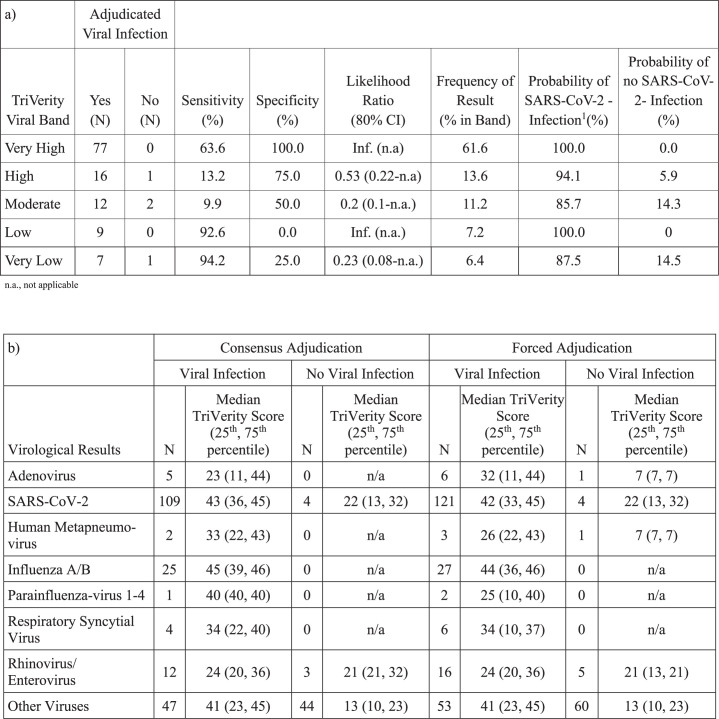
Accuracy of the TriVerity Viral score for the diagnosis of viral infections. **a**, Accuracy of TriVerity for the diagnosis of viral infection in patients positive for SARS-CoV-2. n.a., not applicable. **b**, Median scores observed in patients with specific viruses stratified by viral infection status based on clinical adjudication. n/a, not applicable

**Extended Data Table 5:**
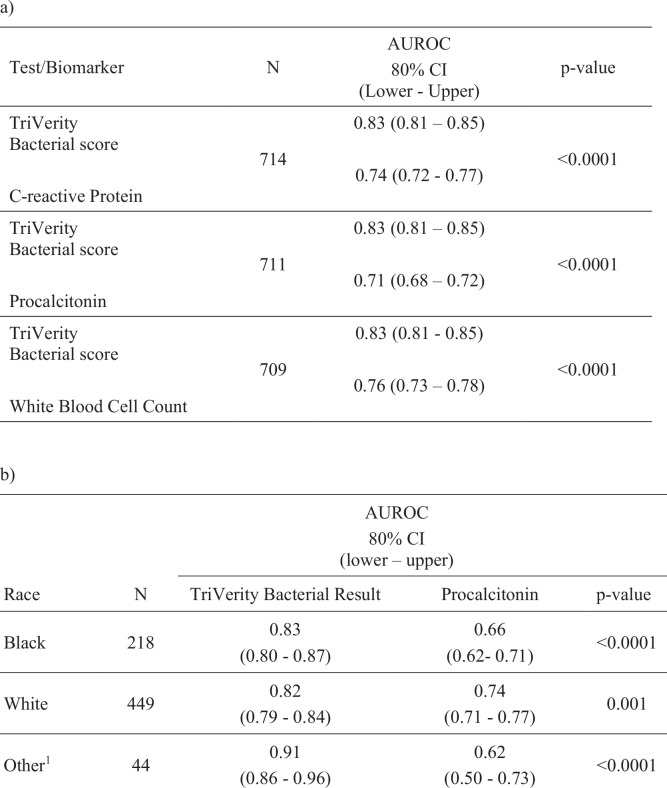
AUROC of the TriVerity Bacterial score for the diagnosis of bacterial infections compared to commonly used biomarkers. (a) Overall AUROC and 80% CI of the TriVerity Bacterial score vs. C-reactive protein, procalcitonin and white blood cell count. (b) Comparison of AUROC of TriVerity Bacterial score vs. procalcitonin across different races

**Extended Data Table 6:**
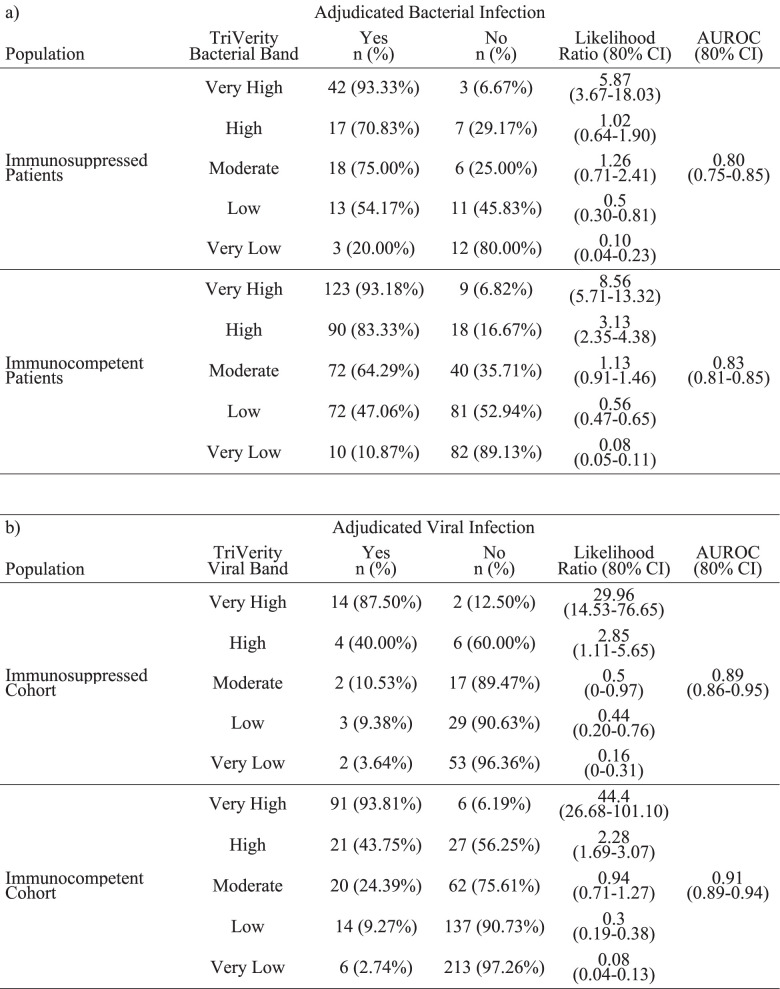
Accuracy of TriVerity Bacterial (**a**) and Viral (**b**) scores stratified by patient immune status

**Extended Data Table 7:**
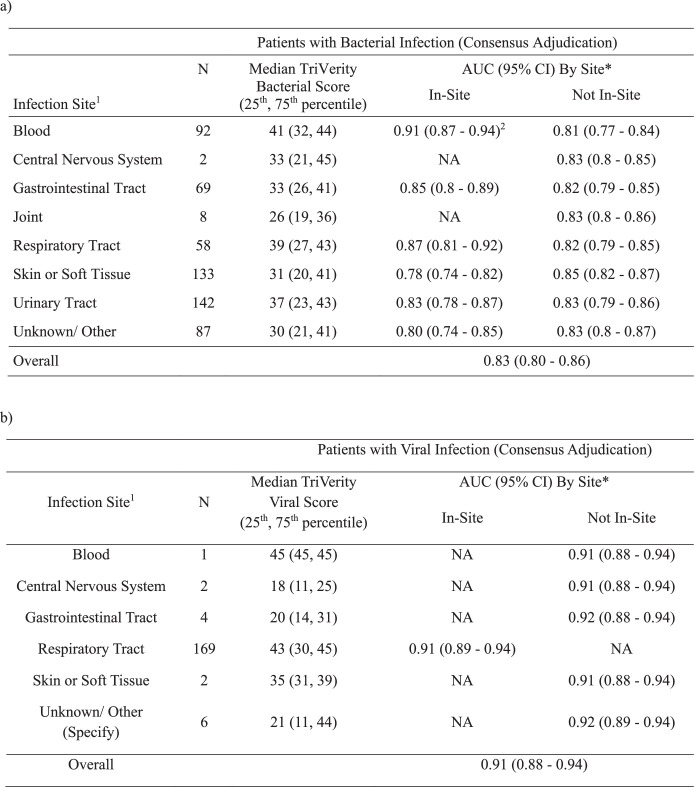
Median TriVerity Bacterial (**a**) and Viral (**b**) scores stratified by anatomical site of infection using consensus adjudication. ^1^ Infection site based on clinical adjudication (more than one site may be listed). ^2^ Only AUCs for the Bacterial scores in patients with bloodstream infections are significantly (*P* < 0.05) higher than the overall bacterial AUCs, likely reflecting the expected more severe and widespread host response for that infection type. All other reported site-specific AUCs do not differ significantly from the overall AUCs. * NA, AUC was not calculated for comparisons with less than 5% cases to avoid high group imbalance

**Extended Data Table 8:**
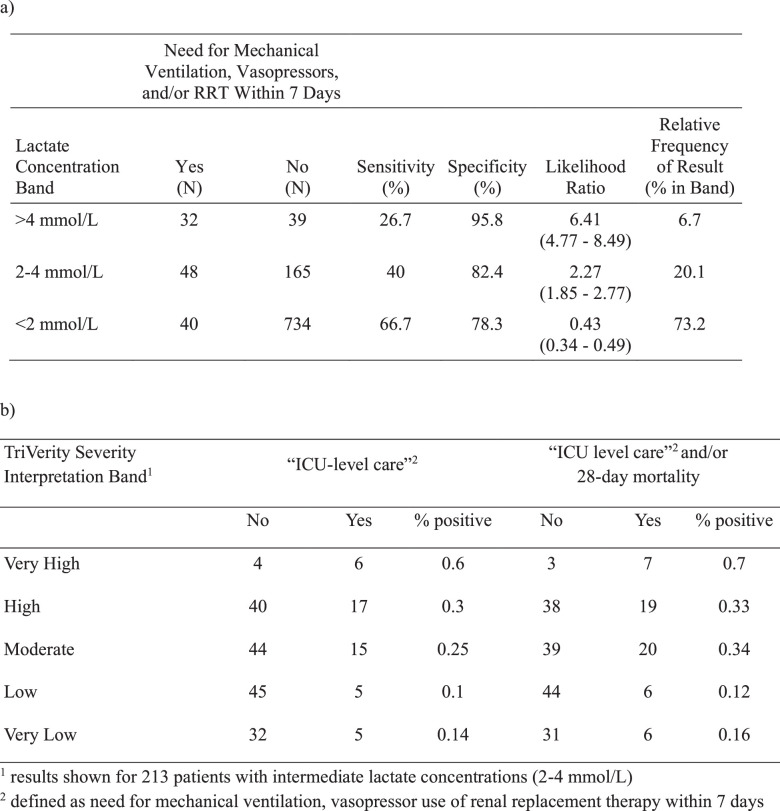
Accuracy of lactate concentration ranges for the prediction of need for mechanical ventilation, vasopressor use and/or RRT within 7 days of presentation in the emergency department. ^1^ results shown for 213 patients with intermediate lactate concentrations (2–4 mmol l^−1^). ^2^ defined as need for mechanical ventilation, vasopressor use and/or RRT within 7 days

**Extended Data Table 9:**
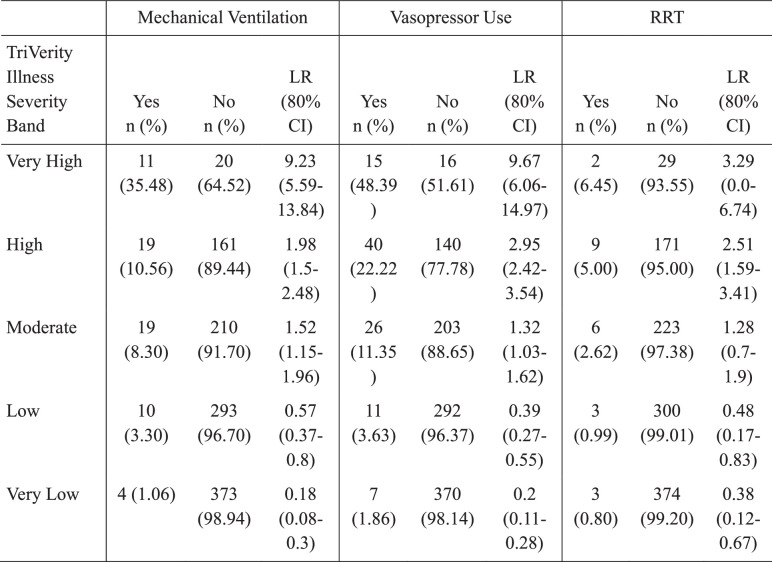
Likelihood ratios of TriVerity Severity score by interpretation band for the prediction of need for mechanical ventilation, vasopressor use and/or RRT within 7 days

## Data Availability

Clinical and other data are stored in a secure database and are owned by Inflammatix, Inc. as the sponsor of the study. Data underlying results of the present study can be shared with academic researchers upon institutional review board approval and subject to intellectual property limitations related to company confidentiality. Please contact clinicaltrials@inflammatix.com. Responses to requests will be sent within 4 weeks.

## Code availability

TriVerity classifier code is not publicly available as it is owned by Inflammatix, Inc. and is part of the locked TriVerity system.

## References

[1] Singer, M. et al. The Third International Consensus Definitions for Sepsis and Septic Shock (Sepsis-3). *JAMA* **315**, 801–810 (2016). 26903338 10.1001/jama.2016.0287 PMC4968574

[2] Evans, L. et al. Surviving sepsis campaign: international guidelines for management of sepsis and septic shock 2021. *Crit. Care Med.* **47**, 1181–1247 (2021). 10.1007/s00134-021-06506-y PMC8486643 34599691

[3] Meyer, N. J. & Prescott, H. C. Sepsis and septic shock. *N. Engl. J. Med.* **391**, 2133–2146 (2024). 39774315 10.1056/NEJMra2403213

[4] Seymour, C. W. et al. Time to treatment and mortality during mandated emergency care for sepsis. *N. Engl. J. Med.* **376**, 2235–2244 (2017). 28528569 10.1056/NEJMoa1703058 PMC5538258

[5] Peltan, I. D. et al. ED door-to-antibiotic time and long-term mortality in sepsis. *Chest* **155**, 938–946 (2019). 30779916 10.1016/j.chest.2019.02.008 PMC6533450

[6] Prescott, H. C. & Iwashyna, T. J. Improving sepsis treatment by embracing diagnostic uncertainty. *Ann. Am. Thorac. Soc.* **16**, 426–429 (2019). 30883190 10.1513/AnnalsATS.201809-646PS PMC6441693

[7] Ducharme, J. et al. A multi-mRNA host-response molecular blood test for the diagnosis and prognosis of acute infections and sepsis: proceedings from a clinical advisory panel. *J. Pers. Med.* **10**, 266 (2020). 10.3390/jpm10040266 PMC7762405 33297498

[8] Gunsolus, I. L., Sweeney, T. E., Liesenfeld, O. & Ledeboer, N. A. Diagnosing and managing sepsis by probing the host response to infection: advances, opportunities, and challenges. *J. Clin. Microbiol.* **57**, e00425-19 (2019). 10.1128/JCM.00425-19 PMC6595443 31043466

[9] Jain, S., Self, W. H., Wunderink, R. G. & CDC EPIC Study Team. Community-acquired pneumonia requiring hospitalization. *N. Engl. J. Med.* **373**, 2382 (2015). 10.1056/NEJMc1511751 PMC9338768 26650159

[10] Ohnuma, T. et al. Association of appropriate empirical antimicrobial therapy with in-hospital mortality in patients with bloodstream infections in the US. *JAMA Netw. Open* **6**, e2249353 (2023). 36598788 10.1001/jamanetworkopen.2022.49353 PMC9857618

[11] Simchen, E. et al. Survival of critically ill patients hospitalized in and out of intensive care. *Crit. Care Med.* **35**, 449–457 (2007). 17167350 10.1097/01.CCM.0000253407.89594.15

[12] Boussina, A. et al. Impact of a deep learning sepsis prediction model on quality of care and survival. *NPJ Digit. Med.* **7**, 14 (2024). 38263386 10.1038/s41746-023-00986-6 PMC10805720

[13] Sundrani, S. et al. Predicting patient decompensation from continuous physiologic monitoring in the emergency department. *NPJ Digit. Med.* **6**, 60 (2023). 37016152 10.1038/s41746-023-00803-0 PMC10073111

[14] He, Y. D. et al. The optimization and biological significance of a 29-host-immune-mRNA panel for the diagnosis of acute infections and sepsis. *J. Pers. Med.* **11**, 735 (2021). 10.3390/jpm11080735 PMC8402342 34442377

[15] Sweeney, T. E., Wong, H. R. & Khatri, P. Robust classification of bacterial and viral infections via integrated host gene expression diagnostics. *Sci. Transl. Med.* **8**, 346ra391 (2016). 10.1126/scitranslmed.aaf7165 PMC5348917 27384347

[16] Sweeney, T. E., Shidham, A., Wong, H. R. & Khatri, P. A comprehensive time-course-based multicohort analysis of sepsis and sterile inflammation reveals a robust diagnostic gene set. *Sci. Transl. Med.* **7**, 287ra271 (2015). 10.1126/scitranslmed.aaa5993 PMC4734362 25972003

[17] Sweeney, T. E. et al. A community approach to mortality prediction in sepsis via gene expression analysis. *Nat. Commun.* **9**, 694 (2018). 29449546 10.1038/s41467-018-03078-2 PMC5814463

[18] Galtung, N. et al. Prospective validation of a transcriptomic severity classifier among patients with suspected acute infection and sepsis in the emergency department. *Eur. J. Emerg. Med.* **29**, 357–365 (2022). 10.1097/MEJ.0000000000000931 PMC9432813 35467566

[19] Bauer, W. et al. A novel 29-messenger RNA host-response assay from whole blood accurately identifies bacterial and viral infections in patients presenting to the emergency department with suspected infections: a prospective observational study. *Crit. Care Med.* **49**, 1664–1673 (2021). 34166284 10.1097/CCM.0000000000005119 PMC8439671

[20] Safarika, A. et al. A 29-mRNA host response test from blood accurately distinguishes bacterial and viral infections among emergency department patients. *Intensive Care Med. Exp.* **9**, 31 (2021). 34142256 10.1186/s40635-021-00394-8 PMC8211458

[21] Kostaki, A. et al. A 29-mRNA host response whole-blood signature improves prediction of 28-day mortality and 7-day intensive care unit care in adults presenting to the emergency department with suspected acute infection and/or sepsis. *Shock* **58**, 224–230 (2022). 36125356 10.1097/SHK.0000000000001970 PMC9512237

[22] Whitfield, N. N. et al. A standardized protocol using clinical adjudication to define true infection status in patients presenting to the emergency department with suspected infections and/or sepsis. *Diagn. Microbiol. Infect. Dis.* **110**, 116382 (2024). 38850687 10.1016/j.diagmicrobio.2024.116382

[23] Taylor, S. P. et al. A quantitative study of decision thresholds for initiation of antibiotics in suspected sepsis. *Med. Decis. Making* **43**, 175–182 (2023). 36062810 10.1177/0272989X221121279

[24] Mi, M. Y., Klompas, M. & Evans, L. Early administration of antibiotics for suspected sepsis. *N. Engl. J. Med.* **380**, 593–596 (2019). 30726686 10.1056/NEJMclde1809210

[25] Carroll, K. C. Assessment of MeMed BV assays for differentiating between bacterial and viral respiratory infections. *Expert Rev. Mol. Diagn.* **24**, 873–884 (2024). 10.1080/14737159.2024.2408743 39314006

[26] Pulia, M., Redwood, R. & May, L. Antimicrobial stewardship in the emergency department. *Emerg. Med. Clin. North Am.* **36**, 853–872 (2018). 30297009 10.1016/j.emc.2018.06.012 PMC7094813

[27] Tamma, P. D., Avdic, E., Li, D. X., Dzintars, K. & Cosgrove, S. E. Association of adverse events with antibiotic use in hospitalized patients. *JAMA Intern. Med.* **177**, 1308–1315 (2017). 28604925 10.1001/jamainternmed.2017.1938 PMC5710569

[28] Dittrich, S. et al. Target product profile for a diagnostic assay to differentiate between bacterial and non-bacterial infections and reduce antimicrobial overuse in resource-limited settings: an expert consensus. *PLoS ONE* **11**, e0161721 (2016). 27559728 10.1371/journal.pone.0161721 PMC4999186

[29] Shapiro, N. I. et al. Diagnostic accuracy of a bacterial and viral biomarker point-of-care test in the outpatient setting. *JAMA Netw. Open* **5**, e2234588 (2022). 36255727 10.1001/jamanetworkopen.2022.34588 PMC9579916

[30] Novak, D. et al. MeMed BV testing in emergency department patients presenting with febrile illness concerning for respiratory tract infection. *Am. J. Emerg. Med.* **65**, 195–199 (2023). 36437179 10.1016/j.ajem.2022.11.022

[31] Ram-Mohan, N. et al. Using a 29-mRNA host response classifier to detect bacterial coinfections and predict outcomes in COVID-19 patients presenting to the emergency department. *Microbiol. Spectr.* **10**, e0230522 (2022). 36250865 10.1128/spectrum.02305-22 PMC9769905

[32] Bauer, W. et al. Detection of viral infection and bacterial coinfection and superinfection in coronavirus disease 2019 patients presenting to the emergency department using the 29-mRNA host response classifier IMX-BVN-3: a multicenter study. *Open Forum Infect. Dis.* **9**, ofac437 (2022). 36111173 10.1093/ofid/ofac437 PMC9452140

[33] Newman-Toker, D. E. & Sharfstein, J. M. The role for policy in AI-assisted medical diagnosis. *JAMA Health Forum* **5**, e241339 (2024). 38635262 10.1001/jamahealthforum.2024.1339 PMC12419546

[34] Rhee, C. et al. Infectious Diseases Society of America position paper: recommended revisions to the national Severe Sepsis and Septic Shock Early Management Bundle (SEP-1) sepsis quality measure. *Clin. Infect. Dis.* **72**, 541–552 (2020). 10.1093/cid/ciaa059 PMC8189682 32374861

[35] Pakyz, A. L. et al. Impact of the Centers for Medicare and Medicaid Services sepsis core measure on antibiotic use. *Clin. Infect. Dis.* **72**, 556–565 (2021). 32827032 10.1093/cid/ciaa456

[36] Freund, Y. et al. Prognostic accuracy of Sepsis-3 criteria for in-hospital mortality among patients with suspected infection presenting to the emergency department. *JAMA* **317**, 301–308 (2017). 28114554 10.1001/jama.2016.20329

[37] Balk, R. et al. Rapid and robust identification of sepsis using SeptiCyte RAPID in a heterogeneous patient population. *J Clin. Med.* **13**, 6044 (2024). 10.3390/jcm13206044 PMC11509035 39457994

[38] Linnander, E. L. et al. Mitigating structural racism to reduce inequities in sepsis outcomes: a mixed methods, longitudinal intervention study. *BMC Health Serv. Res.* **22**, 975 (2022). 35907839 10.1186/s12913-022-08331-5 PMC9338573

[39] van Houten, C. B. et al. A host-protein based assay to differentiate between bacterial and viral infections in preschool children (OPPORTUNITY): a double-blind, multicentre, validation study. *Lancet Infect. Dis.* **17**, 431–440 (2017). 28012942 10.1016/S1473-3099(16)30519-9

[40] Halabi, S. et al. Host test based on tumor necrosis factor-related apoptosis-inducing ligand, interferon gamma-induced protein-10 and C-reactive protein for differentiating bacterial and viral respiratory tract infections in adults: diagnostic accuracy study. *Clin. Microbiol. Infect.* **29**, 1159–1165 (2023). 37270059 10.1016/j.cmi.2023.05.033

[41] Bachur, R. G. et al. A rapid host–protein test for differentiating bacterial from viral infection: Apollo diagnostic accuracy study. *J. Am. Coll. Emerg. Physicians Open* **5**, e13167 (2024). 38721037 10.1002/emp2.13167 PMC11077430

[42] Martin, L. S. et al. Diagnostic accuracy of LIAISON MeMed VB® for bacteremia in the emergency department. Enferm. *Infecc. Microbiol. Clin. (Engl. Ed.)* **43**, 302–303 (2025). 10.1016/j.eimce.2025.01.007 40340039

[43] Buturovic, L. et al. Development of machine learning classifiers for blood-based diagnosis and prognosis of suspected acute infections and sepsis. *Proc. Mach. Learn. Res.* **259**, 154–170 (2025).

[44] Mayhew, M. B. et al. A generalizable 29-mRNA neural-network classifier for acute bacterial and viral infections. *Nat. Commun.* **11**, 1177 (2020). 32132525 10.1038/s41467-020-14975-w PMC7055276

[45] Brakenridge, S. C. et al. A transcriptomic severity metric that predicts clinical outcomes in critically ill surgical sepsis patients. *Crit. Care Explor.* **3**, e0554 (2021). 34671746 10.1097/CCE.0000000000000554 PMC8522866

[46] Brakenridge, S. C. et al. Evaluation of a multivalent transcriptomic metric for diagnosing surgical sepsis and estimating mortality among critically ill patients. *JAMA Netw. Open* **5**, e2221520 (2022). 35819783 10.1001/jamanetworkopen.2022.21520 PMC9277492

[47] Remmel, M. C., Coyle, S. M., Eshoo, M. W., Sweeney, T. E. & Rawling, D. C. Diagnostic host gene expression analysis by quantitative reverse transcription loop-mediated isothermal amplification to discriminate between bacterial and viral infections. *Clin. Chem.* **68**, 550–560 (2022). 35134876 10.1093/clinchem/hvab275

[48] Figueiredo-Pereira, C. et al. Analytical evaluation of TriVerity, a rapid diagnostic and prognostic host gene expression test performed on the Myrna instrument using RT-LAMP. *J. Clin. Microbiol.*10.1128/jcm.00352-25 (2025). 10.1128/jcm.00352-25 PMC12506079 40862618

