# Supplementary material for: Clinical validation of an AI-based blood testing device for diagnosis and prognosis of acute infection and sepsis
Source: Nat Med. 2025 Sep 30;31(12):4044–54. doi: 10.1038/s41591-025-03933-y (PMC12705421; doi:10.1038/s41591-025-03933-y)
Supplement: Supplementary file 1 — Supplementary Tables 1–7 and Supplementary Figs. 1–4. [file 41591_2025_3933_MOESM1_ESM.pdf]

nature medicine

Supplementary information https://doi.org/10.1038/s41591-025-03933-y

Clinical validation of an AI-based blood testing device for diagnosis and prognosis of acute infection and sepsis

In the format provided by the authors and unedited

Supplemental Materials

Supplementary Table 1. Clinical trial sites and number of enrolled patients at each site in patients with consensus and forced adjudication of the infection status and in those evaluable for the prognostic endpoint

	Consensus Adjudication (N=729)	Forced Adjudication (N=1,222)	Prognostic Population (N=1,120)
Site, n (%)			
101 - Baystate Medical Center	62 (8.5%)	90 (7.4%)	83 (7.4%)
102 - Texas Tech University Health Sciences Center - El Paso	55 (7.5%)	69 (5.6%)	67 (6.0%)
103 - University of Kentucky	45 (6.2%)	72 (5.9%)	72 (6.4%)
104 - Henry Ford Hospital	31 (4.3%)	46 (3.8%)	44 (3.9%)
105 - University of Pittsburgh	41 (5.6%)	78 (6.4%)	61 (5.4%)
106 - University of Iowa	97 (13.3%)	168 (13.7%)	153 (13.7%)
107 - Emory University	6 (0.8%)	11 (0.9%)	5 (0.4%)
108 - University of Southern California	8 (1.1%)	14 (1.1%)	14 (1.3%)
109 - Medical College of Wisconsin	50 (6.9%)	87 (7.1%)	83 (7.4%)
110 - Hackensack Meridian Health	2 (0.3%)	2 (0.2%)	2 (0.2%)
111 - MedStar Health Research Institute	11 (1.5%)	16 (1.3%)	15 (1.3%)
112 - Johns Hopkins Hospital	43 (5.9%)	83 (6.8%)	74 (6.6%)
113 - Mayo Clinic - Rochester	75 (10.3%)	124 (10.1%)	118 (10.5%)
114 - Washington University	50 (6.9%)	92 (7.5%)	83 (7.4%)
115 - Vanderbilt University Medical Center	3 (0.4%)	3 (0.2%)	3 (0.3%)
117 - University of South Alabama	1 (0.1%)	1 (0.1%)	0 (0.0%)
118 - University of Athens -Greece	4 (0.5%)	14 (1.1%)	14 (1.3%)
119 - University of California Davis Medical Center	14 (1.9%)	33 (2.7%)	31 (2.8%)
120 - Cleveland Clinic	24 (3.3%)	41 (3.4%)	37 (3.3%)
121 - University of Florida (UF) - Jacksonville	92 (12.6%)	147 (12.0%)	133 (11.9%)
125 - Geisinger Health	5 (0.7%)	9 (0.7%)	8 (0.7%)
135 – Beth Israel Deaconess Medical Center	10 (1.4%)	22 (1.8%)	20 (1.8%)

Supplementary Table 2. Results of laboratory tests in participants at the time of enrollment. a, white blood cell counts or percentages; b, C-reactive protein, procalcitonin and lactate concentrations

a)

White Blood Cell Counts	Diagnostic Population: Consensus (N=729)	Diagnostic Population: Forced (N=1,222)	Prognostic Population (N=1,120)
Eosinophils (%)			
N	653	1087	995
Mean (SD)	0.8 (1.26)	1.0 (1.68)	1.0 (1.72)
Median (Range)	0.3 (0.0, 10.0)	0.4 (0.0, 24.0)	0.4 (0.0, 24.0)
Leukocytes (10^9/L)			
N	709	1179	1081
Mean (SD)	11.8 (6.19)	11.7 (6.06)	11.7 (6.07)
Median (Range)	10.9 (0.7, 41.4)	10.7 (0.7, 41.4)	10.7 (0.7, 41.4)
Lymphocytes (%)			
N	689	1140	1046
Mean (SD)	12.5 (9.26)	13.7 (10.21)	13.5 (10.21)
Median (Range)	10.0 (0.0, 53.7)	11.1 (0.0, 72.0)	11.0 (0.0, 72.0)
Monocytes (%)			
N	688	1139	1045
Mean (SD)	7.3 (3.84)	7.6 (4.47)	7.6 (4.56)
Median (Range)	7.0 (0.0, 44.9)	7.0 (0.0, 60.9)	7.0 (0.0, 60.9)
Neutrophils (%)			
N	687	1138	1045
Mean (SD)	76.9 (12.91)	75.7 (13.60)	75.8 (13.76)
Median (Range)	79.2 (3.0, 98.2)	78.0 (3.0, 98.2)	78.0 (3.0, 98.2)
Neutrophils Bands (%)			
N	138	192	183
Mean (SD)	6.6 (12.13)	6.2 (12.10)	6.0 (11.90)
Median (Range)	1.1 (0.0, 92.0)	1.0 (0.0, 92.0)	1.0 (0.0, 92.0)

b)

C-Reactive Protein, Procalcitonin and Lactate Concentrations	Diagnostic Population: Consensus (N=729)	Diagnostic Population: Forced (N=1222)	Prognostic Population (N=1120)
C-Reactive Protein (mg/dL)			
N	714	1200	1100
Mean (SD)	10.3 (10.39)	8.9 (9.65)	9.1 (9.70)
Median (Range)	6.7 (0.2, 49.4)	5.3 (0.1, 49.4)	5.4 (0.1, 49.4)
Procalcitonin (ng/mL)			
N	711	1186	1091
Mean (SD)	4.6 (16.69)	3.0 (13.14)	3.2 (13.55)
Median (Range)	0.2 (0.0, 238.4)	0.2 (0.0, 238.4)	0.2 (0.0, 238.4)
Lactate (mmol/L)			
N	702	1155	1058
Mean (SD)	1.8 (1.51)	1.9 (1.61)	1.9 (1.66)
Median (Range)	1.4 (0.4, 16.0)	1.5 (0.4, 16.0)	1.4 (0.4, 16.0)

Supplementary Table 3. Type of infection, anatomical localization of infection and AUCs for the bacterial and viral TriVerity Test results compared to standard of care biomarkers in patient with forced adjudication (entire population including "uncertain" cases adjudicated as probable and unlikely infected). a) type and anatomical localization of infection. b) AUC of bacterial TriVerity results compared to C-reactive protein, procalcitonin and white blood cell count

a)	Type of Infection/ Anatomical Location of Infection[2]	% (n/N)
Type of Infection	Bacterial Infection	58.1% (710/1222)
	Viral Infection	20.0% (244/1222)
	Viral-Bacterial Coinfection	2.5% (31/1222)
	Noninfected	19.4% (237/1222)
Bacterial Adjudication[1]	Blood	14.0% (104/741)
	Central Nervous System	0.4% (3/741)
	Gastrointestinal Tract	13.5% (100/741)
	Joint	1.2% (9/741)
	Respiratory Tract	16.7% (124/741)
	Skin or Soft Tissue	33.3% (247/741)
	Urinary Tract	26.9% (199/741)
	Unknown/ Other	19.2% (142/741)

Viral Adjudication[1]	Blood	0.4% (1/275)
	Central Nervous System	1.8% (5/275)
	Gastrointestinal Tract	8.7% (24/275)
	Respiratory Tract	85.1% (234/275)
	Skin or Soft Tissue	2.2% (6/275)
	Unknown/ Other	7.3% (20/275)
Bacterial and/or Viral Adjudication	Blood	8.6% (105/1222)
	Central Nervous System	0.7% (8/1222)
	Gastrointestinal Tract	10.1% (124/1222)
	Joint	0.7% (9/1222)
	Respiratory Tract	29.3% (358/1222)
	Skin or Soft Tissue	20.7% (253/1222)
	Urinary Tract	16.3% (199/1222)
	Unknown/ Other	13.3% (162/1222)

[1] Participants may have multiple sources

[2] Anatomical location of infection was determined by clinical adjudicators after review of the clinical adjudication report

<table>
<tr><td colspan="2">b)</td><td>Comparator</td><td>N</td><td>AUROC</td><td>80% CI</td></tr>
<tr><td>Population/Endpoint</td><td colspan="5">Diagnostic Results</td></tr>
<tr><td rowspan="2">Forced Adjudication</td><td rowspan="2">C-Reactive Protein</td><td>CRP</td><td rowspan="2">1200</td><td>0.73</td><td>0.71 – 0.75</td></tr>
<tr><td>TriVerity Bacterial</td><td>0.77</td><td>0.75 – 0.78</td></tr>
<tr><td rowspan="2">Forced Adjudication</td><td rowspan="2">Procalcitonin</td><td>PCT</td><td rowspan="2">1186</td><td>0.69</td><td>0.67 – 0.71</td></tr>
<tr><td>TriVerity Bacterial</td><td>0.77</td><td>0.75 – 0.78</td></tr>
<tr><td rowspan="2">Forced Adjudication</td><td rowspan="2">White Blood Cells</td><td>WBC</td><td rowspan="2">1179</td><td>0.70</td><td>0.68 – 0.72</td></tr>
<tr><td>TriVerity Bacterial</td><td>0.76</td><td>0.75 – 0.78</td></tr>
</table>

Supplementary Table 4. Cross-classification of TriVerity Bacterial and Viral scores stratified by clinically adjudicated infections status (consensus adjudication). (a) adjudicated bacterial infection, (b) adjudicated viral infection, (c) adjudicated bacterial-viral co-infection, d) adjudicated non-infectious status

a)

	TriVerity Bacterial Score				
TriVerity Viral Score	Very High (N=159)	High (N=105)	Moderate (N=89)	Low (N=83)	Very Low (N=12)
Very High	0 (0.0%)	0 (0.0%)	1 (1.1%)	1 (1.2%)	3 (25.0%)
High	0 (0.0%)	0 (0.0%)	9 (10.1%)	10 (12.0%)	3 (25.0%)
Moderate	3 (1.9%)	17 (16.2%)	23 (25.8%)	19 (22.9%)	3 (25.0%)
Low	34 (21.4%)	50 (47.6%)	31 (34.8%)	23 (27.7%)	0 (0.0%)
Very Low	122 (76.7%)	38 (36.2%)	25 (28.1%)	30 (36.1%)	3 (25.0%)

b)

	TriVerity Bacterial Score				
TriVerity Viral Score	Very High (N=6)	High (N=9)	Moderate (N=20)	Low (N=50)	Very Low (N=80)
Very High	0 (0.0%)	0 (0.0%)	1 (5.0%)	26 (52.0%)	77 (96.3%)
High	0 (0.0%)	2 (22.2%)	8 (40.0%)	12 (24.0%)	1 (1.3%)
Moderate	0 (0.0%)	4 (44.4%)	5 (25.0%)	7 (14.0%)	2 (2.5%)
Low	4 (66.7%)	1 (11.1%)	5 (25.0%)	4 (8.0%)	0 (0.0%)
Very Low	2 (33.3%)	2 (22.2%)	1 (5.0%)	1 (2.0%)	0 (0.0%)

c)

	TriVerity Bacterial Score				
TriVerity Viral Score	Very High (N=6)	High (N=2)	Moderate (N=1)	Low (N=2)	Very Low (N=1)
Very High	0 (0.0%)	0 (0.0%)	0 (0.0%)	0 (0.0%)	1 (100.0%)
High	0 (0.0%)	0 (0.0%)	0 (0.0%)	2 (100.0%)	0 (0.0%)
Moderate	3 (50.0%)	1 (50.0%)	0 (0.0%)	0 (0.0%)	0 (0.0%)
Low	2 (33.3%)	1 (50.0%)	0 (0.0%)	0 (0.0%)	0 (0.0%)
Very Low	1 (16.7%)	0 (0.0%)	1 (100.0%)	0 (0.0%)	0 (0.0%)

d)

TriVerity Viral Score	TriVerity Bacterial Score Very High (N=6)	High (N=16)	Moderate (N=26)	Low (N=42)	Very Low (N=14)
Very High	0 (0.0%)	0 (0.0%)	0 (0.0%)	2 (4.8%)	1 (7.1%)
High	0 (0.0%)	0 (0.0%)	0 (0.0%)	6 (14.3%)	5 (35.7%)
Moderate	0 (0.0%)	2 (12.5%)	2 (7.7%)	9 (21.4%)	1 (7.1%)
Low	0 (0.0%)	3 (18.8%)	5 (19.2%)	15 (35.7%)	5 (35.7%)
Very Low	6 (100.0%)	11 (68.8%)	19 (73.1%)	10 (23.8%)	2 (14.3%)

Supplementary Table 5. Pre- and post-test probabilities for the need for "ICU-level care" within 7 days stratified by qSOFA scores of 0-1 (low risk) or 2-3 (high risk) and TriVerity Severity interpretation band[1]

<table>
<tr><th colspan="6">Patients with Need of "ICU-Level Care"[2] Within 7 Days</th></tr>
<tr><th>Pre-Test Probability</th><th>Post- qSOFA Probability Stratified by Score on Day 0</th><th>Stratified by TriVerity Severity Band</th><th>No</th><th>Yes</th><th>Post-Test Probability</th></tr>
<tr><td rowspan="10">10.9% (122/1,120)</td><td rowspan="5">qSOFA Score 0-1
(Low Risk)
7.71% (74/960)</td><td>Very High</td><td>11</td><td>12</td><td>52.17%</td></tr>
<tr><td>High</td><td>127</td><td>21</td><td>14.19%</td></tr>
<tr><td>Moderate</td><td>159</td><td>24</td><td>13.11%</td></tr>
<tr><td>Low</td><td>249</td><td>10</td><td>3.86%</td></tr>
<tr><td>Very Low</td><td>340</td><td>7</td><td>2.02%</td></tr>
<tr><td rowspan="5">qSOFA Score 2-3
(High Risk)
46.34% (38/82)</td><td>Very High</td><td>2</td><td>5</td><td>71.43%</td></tr>
<tr><td>High</td><td>6</td><td>16</td><td>72.73%</td></tr>
<tr><td>Moderate</td><td>16</td><td>12</td><td>42.86%</td></tr>
<tr><td>Low</td><td>14</td><td>3</td><td>17.65%</td></tr>
<tr><td>Very Low</td><td>6</td><td>2</td><td>25.00%</td></tr>
</table>

[1] 78 patients with incomplete qSOFA scores were excluded from this analysis

[2] "ICU-level care": Need for mechanical ventilation, vasopressor use, and/or renal replacement therapy

Supplementary Table 6. Accuracy of the TriVerity severity results for the prediction of need for mechanical ventilation, vasopressor use and/or renal replacement therapy within 7 days and/or 28-day mortality stratified by interpretation band

	Need for "ICU-level care"[1] and/or 28-day mortality							
**TriVerity Illness Severity band**	**Yes (n)**	**No (n)**	**Sensiti-vity (%)**	**Specifi-city (%)**	**Likelihood ratio**	**Frequency of result (% in band)**	**Probability of ICU-level care* and/or 28-day mortality (%)**	**Probability of no ICU-level care* and/or 28-day survival (%)**
Very High	21	9	14.3	99.0	14.86	2.8	70	30
High	46	127	31.3	86.4	2.31	16	26.6	73.4
Moderate	47	171	32.0	81.7	1.75	20.1	21.6	78.4
Low	19	272	87.1	29.1	0.44	26.9	6.5	93.5
Very Low	14	357	90.5	38.1	0.25	34.3	3.8	96.2

[1]defined as the prediction of need for mechanical ventilation, vasopressor use and/or renal replacement therapy within 7 days

Supplementary Table 7. Logistic regression model[1] of TriVerity Bacterial score impact vs. other biomarkers and clinical variables in consensus population

Variable	p value	Coefficient	Std. Error
TriVerity Bacterial score	2.03E-10	0.074	0.012
WBC	0.002	0.079	0.023
PCT	0.006	0.301	0.108
CRP	0.02	0.034	0.015
SEX	0.01	-0.521	0.207
AGE	0.05	-0.012	0.006
RACE	NS	0.035	0.227

[1]Bacterial adjudication status was used as the independent variable (with bacterial and co-infected samples coded as "Yes" and viral and non-infected as "No"), and TriVerity Bacterial score was used as predictor along with PCT, CRP, WBC count and demographic variables. Only samples with complete data were used (N=681). Even though adjudicators were presented with all the analyzed variables except TriVerity Bacterial score, the score was the most significant variable associated with the outcome (2.03E-10) followed by WBC (0.002) and PCT (0.006). AUC of combined model for all variables was 0.86, while omitting TriVerity Bacterial score resulted in 3-point reduction in AUC. In contrast, AUC of models where either WBC or PCT are omitted was 0.85 showing that TriVerity Bacterial score adds overall more relevant information than any other marker. Also notable, TriVerity Bacterial score by itself, without any laboratory results, produces AUC of 0.83 which is equivalent to the model using all other biomarkers without the Bacterial score.

Supplementary Figures

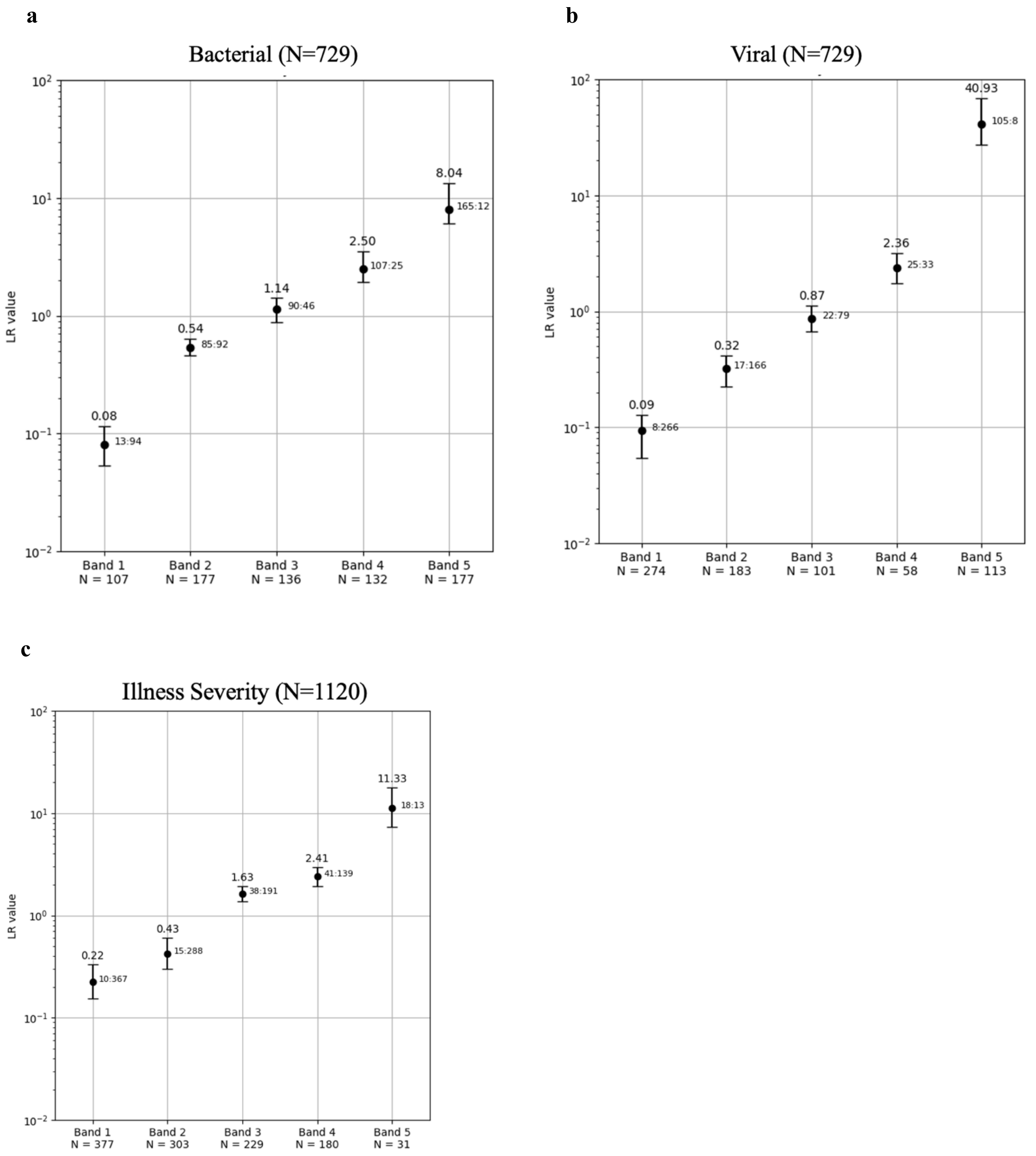

Supplementary Figure 1 | TriVerity likelihood ratios by interpretation band for the diagnosis of bacterial (a) and viral (b) infections, and for the prediction of illness severity (c). Whiskers represent 80% CIs.

a)

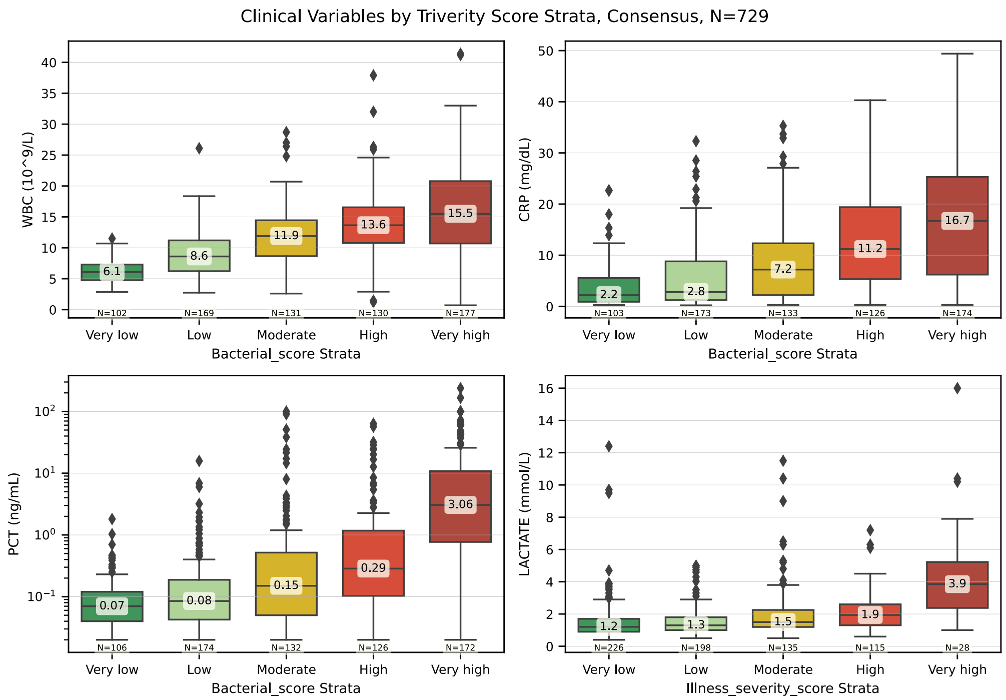

b)

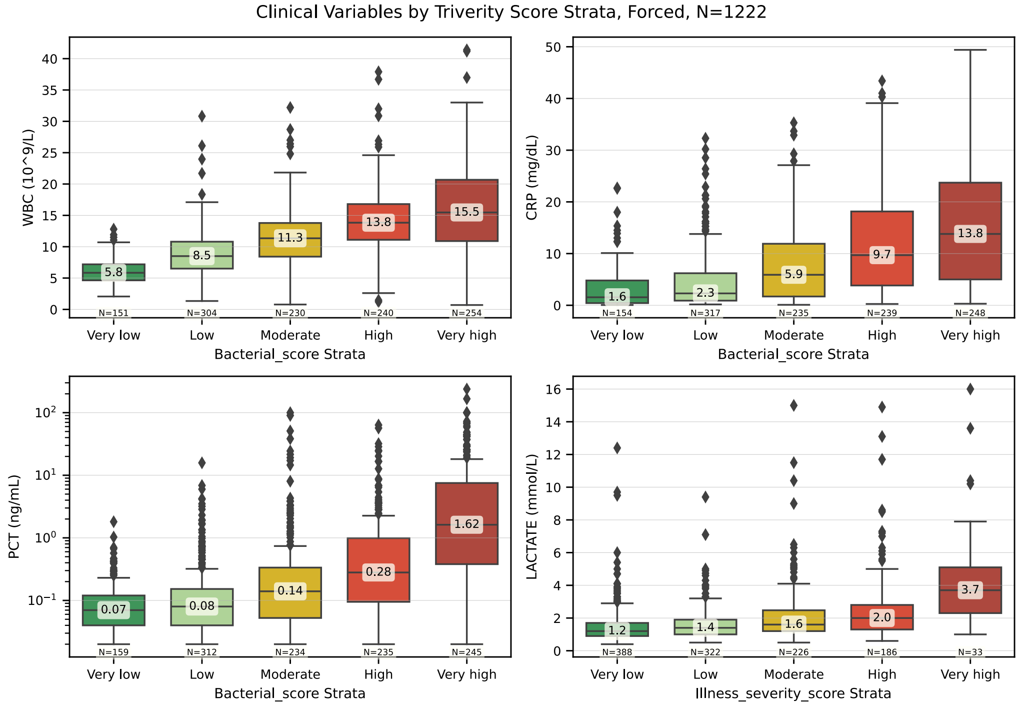

Supplementary Figure 2 | Concentrations of clinically used biomarkers WBC, CRP, PCT and lactate stratified by TriVerity Bacterial or Severity score band. a. Consensus adjudication cohort; b. Forced adjudication cohort

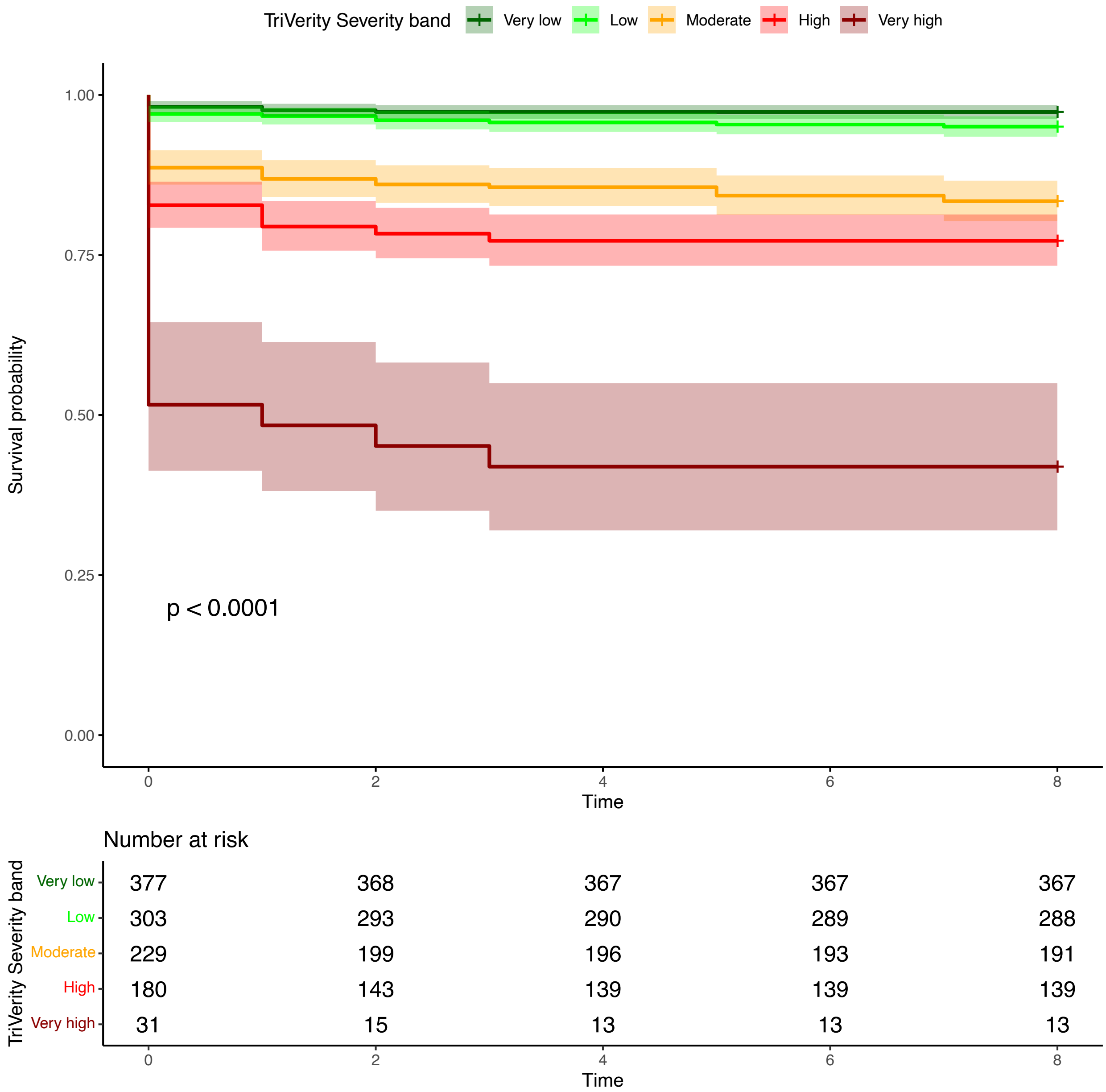

Supplementary Figure 3 | Kaplan-Meier curves depicting probabilities of survival between days 0 and 7 stratified by TriVerity severity interpretation band. Figure presents Kaplan-Meier plot of proportion of patients requiring ICU level care within 7 days from presentation in ED. Shaded areas represent 80% confidence interval. Cox hazard ratio (HR) was calculated relative to patients with TriVerity Severity score in the "Very Low" band.

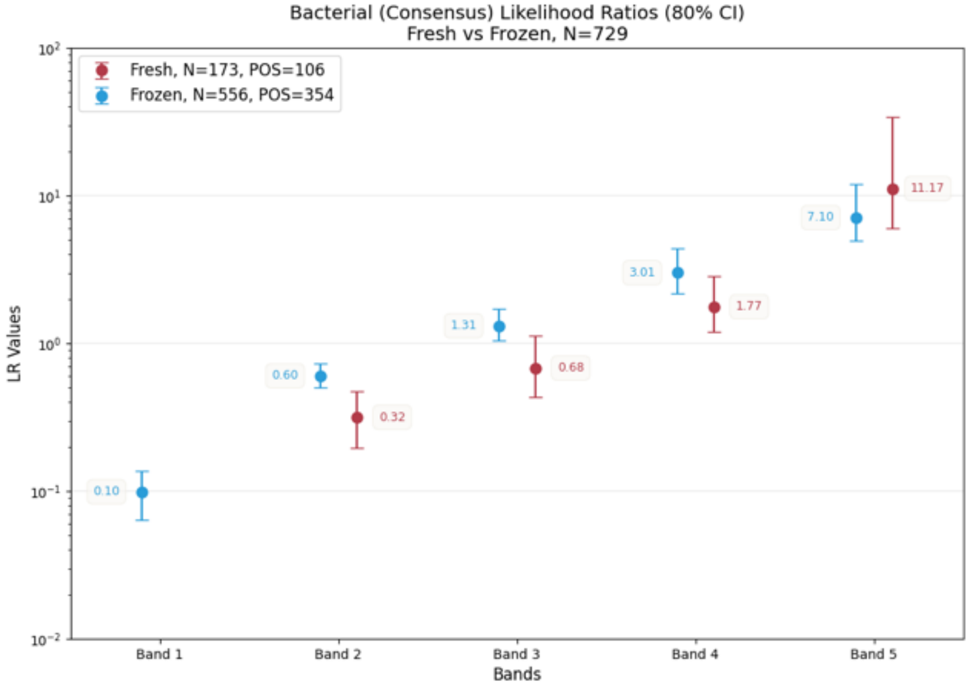
Bacterial (Consensus) Likelihood Ratios (80% CI)
Fresh vs Frozen, N=729
Fresh, N=173, POS=106
Frozen, N=556, POS=354
LR Values
Bands
Band 1
Band 2
Band 3
Band 4
Band 5
0.10
0.60
0.32
1.31
0.68
3.01
1.77
7.10
11.17

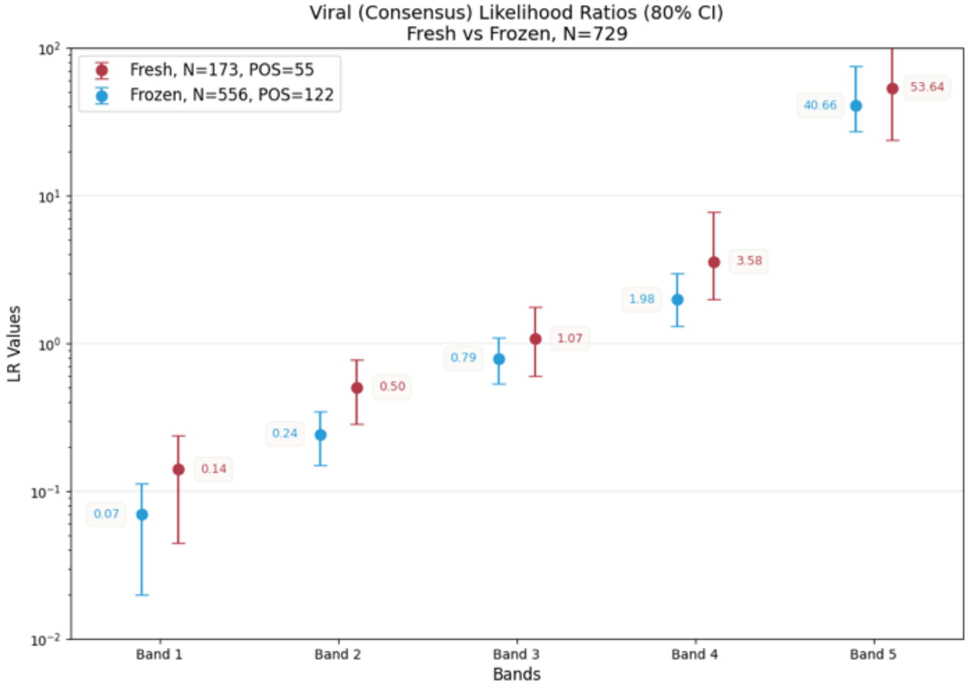
Viral (Consensus) Likelihood Ratios (80% CI)
Fresh vs Frozen, N=729
Fresh, N=173, POS=55
Frozen, N=556, POS=122
LR Values
Bands
Band 1
Band 2
Band 3
Band 4
Band 5
0.07
0.14
0.24
0.50
0.79
1.07
1.98
3.58
40.66
53.64

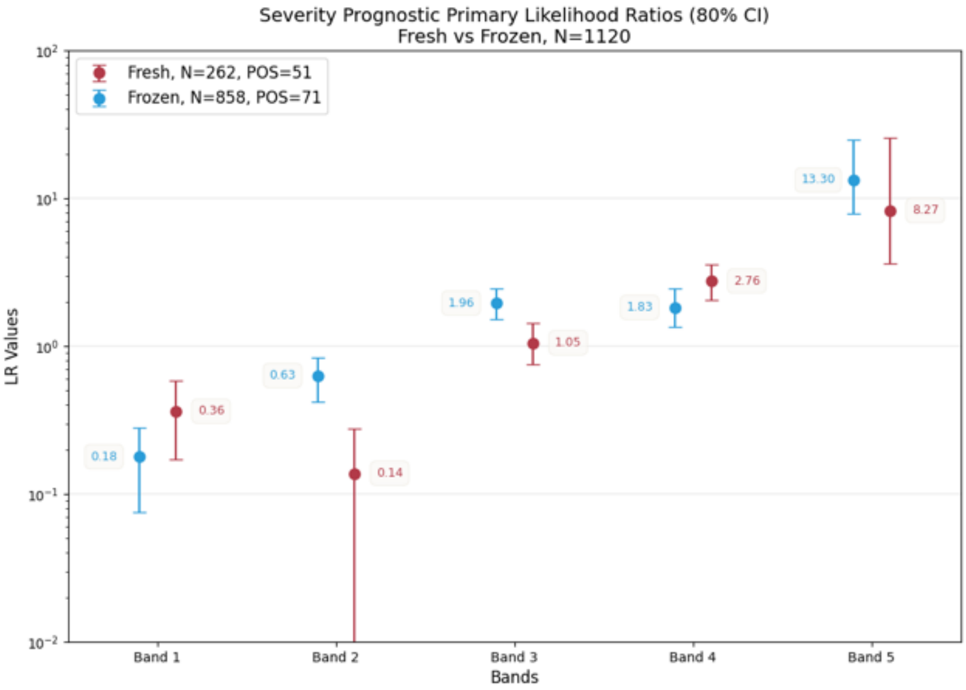

Supplementary Figure 4 | Likelihood ratios of TriVerity scores in fresh and frozen phases of the study. Likelihood ratios and 80% CI for each band and each TriVerity score in subjects recruited during fresh or frozen phases were calculated separately. Bacterial and Viral scores were assessed among the consensus adjudication population, Severity score was assessed on primary prognostic population.
