# Supplementary material for: Clinical validation of an AI-based blood testing device for diagnosis and prognosis of acute infection and sepsis
Source: Nat Med. 2025 Sep 30;31(12):4044–54. doi: 10.1038/s41591-025-03933-y (PMC12705421; doi:10.1038/s41591-025-03933-y)
Supplement: Supplementary file 2 — Reporting Summary [file 41591_2025_3933_MOESM2_ESM.pdf]

nature portfolio

Corresponding author(s): Liesenfeld, O.

Last updated by author(s): May 25, 2025

Reporting Summary

Nature Portfolio wishes to improve the reproducibility of the work that we publish. This form provides structure for consistency and transparency in reporting. For further information on Nature Portfolio policies, see our Editorial Policies and the Editorial Policy Checklist.

Statistics

For all statistical analyses, confirm that the following items are present in the figure legend, table legend, main text, or Methods section.

n/a	Confirmed	
☐	☒	The exact sample size (*n*) for each experimental group/condition, given as a discrete number and unit of measurement
☐	☒	A statement on whether measurements were taken from distinct samples or whether the same sample was measured repeatedly
☐	☒	The statistical test(s) used AND whether they are one- or two-sided<br>*Only common tests should be described solely by name; describe more complex techniques in the Methods section.*
☐	☒	A description of all covariates tested
☐	☒	A description of any assumptions or corrections, such as tests of normality and adjustment for multiple comparisons
☐	☒	A full description of the statistical parameters including central tendency (e.g. means) or other basic estimates (e.g. regression coefficient) AND variation (e.g. standard deviation) or associated estimates of uncertainty (e.g. confidence intervals)
☐	☒	For null hypothesis testing, the test statistic (e.g. *F*, *t*, *r*) with confidence intervals, effect sizes, degrees of freedom and *P* value noted<br>*Give P values as exact values whenever suitable.*
☐	☒	For Bayesian analysis, information on the choice of priors and Markov chain Monte Carlo settings
☒	☐	For hierarchical and complex designs, identification of the appropriate level for tests and full reporting of outcomes
☒	☐	Estimates of effect sizes (e.g. Cohen's *d*, Pearson's *r*), indicating how they were calculated

Our web collection on statistics for biologists contains articles on many of the points above.

Software and code

Policy information about availability of computer code

Data collection	Data were collected from EHRs at enrolling sites and from laboratory results at central laboratories and entered into a secure Medrio database for statistical analysis as stated in the Methods section
Data analysis	SAS and R were used as stated in Methods section, incl version numbers

For manuscripts utilizing custom algorithms or software that are central to the research but not yet described in published literature, software must be made available to editors and reviewers. We strongly encourage code deposition in a community repository (e.g. GitHub). See the Nature Portfolio guidelines for submitting code & software for further information.

Data

Policy information about availability of data

All manuscripts must include a data availability statement. This statement should provide the following information, where applicable:

- Accession codes, unique identifiers, or web links for publicly available datasets
- A description of any restrictions on data availability
- For clinical datasets or third party data, please ensure that the statement adheres to our policy

Clinical and other data are stored in a secure database and owned by Inflammatix as the sponsor of the study. Data underlying results of the present study can be shared with academic researchers upon IRB approval and subject to limitations on IP-related company confidentiality". Please contact clinicaltrials@inflammatix.com. Responses to requests will be sent within 4 weeks.

Research involving human participants, their data, or biological material

Policy information about studies with human participants or human data. See also policy information about sex, gender (identity/presentation), and sexual orientation and race, ethnicity and racism.

Reporting on sex and gender	All-comers were enrolled from 22 EDs across diverse geographiies. Representation of sex, gender, race etc. is a critical part of the study and stated in the manuscript.
Reporting on race, ethnicity, or other socially relevant groupings	Race, gender and sex were recorded in CRFs constructed with clinical sites.
Population characteristics	Other detailed population characteristics incl. relevant co-variates are shown throughout the manuscript, ie Extednded Data Table 1 and Suppl. Table 2
Recruitment	Participants were recruited (all-comers) at clinical sites using electronic and other screening tools. Consent was obtained after IRB approvals. There was no self-collection bias.
Ethics oversight	This study was approved by each local enrolling site, or a central Institutional Review Boards (Advarra).

Note that full information on the approval of the study protocol must also be provided in the manuscript.

Field-specific reporting

Please select the one below that is the best fit for your research. If you are not sure, read the appropriate sections before making your selection.

☒ Life sciences ☐ Behavioural & social sciences ☐ Ecological, evolutionary & environmental sciences

For a reference copy of the document with all sections, see nature.com/documents/nr-reporting-summary-flat.pdf

Life sciences study design

All studies must disclose on these points even when the disclosure is negative.

Sample size	Sample size was determined by statistical power analysis in accordance with FDA guidance.
Data exclusions	o data were excluded other than from participants that did not meet I/E criteria or withdrew from the study, or had invalid test results as described in the Methods section
Replication	this is a large registrational trial conducted in agreement with FDA for clearance of the diagnostic test. Enrolling sites and central laboratories were audited (Source Data verification from EHRs) and 100% of data were replicated/controlled to ensure complete and clean data entry.
Randomization	The manuscript describes results of a non-interventional trial conducted for FDA clearance of a diagnostic test. Clearance is the first path for commercialization of test, interventional trials that required randomization will be conducted in the future.
Blinding	Clinical adjudicators who determined the gold standard/ground truth of patient infection status were blinded to results for the TriVerity test at all times.

Reporting for specific materials, systems and methods

We require information from authors about some types of materials, experimental systems and methods used in many studies. Here, indicate whether each material, system or method listed is relevant to your study. If you are not sure if a list item applies to your research, read the appropriate section before selecting a response.

Materials & experimental systems

n/a	Involved in the study	
☒	☐	Antibodies
☒	☐	Eukaryotic cell lines
☒	☐	Palaeontology and archaeology
☒	☐	Animals and other organisms
☐	☒	Clinical data
☒	☐	Dual use research of concern
☒	☐	Plants

Methods

n/a	Involved in the study	
☒	☐	ChIP-seq
☒	☐	Flow cytometry
☒	☐	MRI-based neuroimaging

Clinical data

Policy information about clinical studies

All manuscripts should comply with the ICMJE guidelines for publication of clinical research and a completed CONSORT checklist must be included with all submissions.

Clinical trial registration	clinicaltrials.gov: NCT04094818
Study protocol	Trial protocol details were shared on clinicaltrials.gov and are provided in the Methods section
Data collection	At all enrolling Emergency Departments, trained and licensed clinical research staff collected participant data from EHRs starting at the time of ED presentation (and 7 days prior) until d28 (when available), a phone f/u was conducted in participants lost to f/up during the 28 day period. Laboratory sites collected data throughout the study period as well. The study collected data between 02 March 2020 to 16 February 2023 and between 08 December 2023 to 28 May 2024.
Outcomes	Primary and secondary clinical outcomes were predefined, and agreed uponi with FDA): primary outcomes were a) the patient infection status as determined post-hoc by clinical adjudication and b) the need for 7 day ICU level care. Secondary outcome was the need for 7 day ICU level care and/or 28-day mortality (both outcomes are described in detail in Methods).

Plants

Seed stocks	n/a
Novel plant genotypes	n/a
Authentication	n/a
